# Pharmacological cholesterol depletion disturbs ciliogenesis and ciliary function in developing zebrafish

**DOI:** 10.1038/s42003-018-0272-7

**Published:** 2019-01-29

**Authors:** Lars D. Maerz, Martin D. Burkhalter, Carolin Schilpp, Oliver H. Wittekindt, Manfred Frick, Melanie Philipp

**Affiliations:** 1 Institute of Biochemistry and Molecular Biology, Ulm University, Albert-Einstein-Allee 11, 89081 Ulm, Germany; 2 Institute of General Physiology, Ulm University, Albert-Einstein-Allee 11, 89081 Ulm, Germany

## Abstract

Patients with an inherited inability to synthesize sufficient amounts of cholesterol develop congenital malformations of the skull, toes, kidney and heart. As development of these structures depends on functional cilia we investigated whether cholesterol regulates ciliogenesis through inhibition of hydroxymethylglutaryl-Coenzyme A reductase (HMG-CoA-R), the rate-limiting enzyme in cholesterol synthesis. HMG-CoA-R is efficiently inhibited by statins, a standard medication for hyperlipidemia. When zebrafish embryos are treated with statins cilia dysfunction phenotypes including heart defects, left-right asymmetry defects and malformation of ciliated organs develop, which are ameliorated by cholesterol replenishment. HMG-CoA-R inhibition and other means of cholesterol reduction lowered ciliation frequency and cilia length in zebrafish as well as several mammalian cell types. Cholesterol depletion further triggers an inability for ciliary signalling. Because of a reduction of the transition zone component Pi(4,5)P_2_ we propose that cholesterol governs crucial steps of cilium extension. Taken together, we report that cholesterol abrogation provokes cilia defects.

## Abstract (web summary)

Lars Maerz et al. show that pharmacological inhibition of cholesterol synthesis in zebrafish embryos leads to organ malformations and dysfunctions in cilium formation and signaling. These findings together with alterations to the cilia transition zone suggest an important role of cholesterol in cilia biogenesis and function.

## Introduction

Despite the appearance of new treatment options, hydroxymethylglutaryl-coenzyme A reductase (HMG-CoA-R) inhibitors, better known as statins, remain the gold standard in lipid lowering therapy. Interestingly, statins are often administered in very high doses, which greatly exceed the actual effective dose 50 (ED_50_) and hence provoke a number of adverse effects^1^. Additionally, statins have been discussed as potentially teratogenic, which appears plausible considering the dependence of the developing foetus on endogenous as well as maternal cholesterol^2^, but has also remained controversial because clinical studies have come to diverging conclusions^3–5^. In the light of the clinical manifestations of low cholesterol syndromes like Smith–Lemli–Opitz syndrome (SLOS), however, we decided to investigate further the impact of lipid lowering therapy during embryogenesis. SLOS patients suffer from congenital multi-organ malformations including growth retardation, microcephaly, facial dysmorphologies, 2–3 syndactyly of the toes and ambiguous genitalia^6^. In addition, congenital heart defects (CHD) and renal malformations such as kidney cysts occur in a substantial number of all SLOS patients^6^. These symptoms together with recurrent respiratory infections are characteristic for diseases, which are caused by impaired cilia formation or function^7^. Moreover, disturbance in signalling cascades, which rely on functional cilia such as Hedgehog signalling, lead to very similar malformations including heart defects^8,9^.

Cilia are hair-like protrusions which emanate from cells not undergoing active division. They consist of a tubulin scaffold, the so-called axoneme, which is ensheathed by a membrane that is distinct from the plasma membrane, but contains high amounts of cholesterol just as it can be found in lipid rafts, too^10,11^. These raft-like accumulations in the membrane of cilia and flagella have been reported crucial for signal transduction such as Hedgehog signalling and hence cilia function^12–14^. In addition to signal transduction, cilia execute a multitude of other sensory and mechanic processes from signal or flow sensing to propulsion of certain body fluids in cavities such as the brain ventricles. Hence, cilia dysfunction is causal for many afflictions ranging from congenital malformations to respiratory impairments, polycystic organs, obesity and even neurological disorders^7,8^. In addition, a growing number of studies suggest that syndromic as well as isolated congenital heart defects can be caused by cilia deficiencies^15,16^. Because of the importance of cilia during development and the fact that cholesterol is an integral component of ciliary membranes, we analysed in the current study whether statin treatment impacts on cilia. Here, we describe that treatment of zebrafish embryos with different statins results in congenital malformations, which largely resemble not only the morphological characteristics of SLOS, but also the clinical appearance of ciliopathies. Statin treatment provokes defects in cilium formation and hence cilium dysfunction in developing zebrafish. Such cilium aberrations can also be observed in different mammalian cell culture models and the evolutionarily low ciliate *Tetrahymena thermophila*. Importantly, other means of cholesterol depletion as well as reconstitution experiments using soluble cholesterol suggest that phenotypes induced by statin treatment arise from low cholesterol abundance. Mechanistically, the observed cilium defects may arise from alterations to the transition zone of the cilium which lacks Pi(4,5)P_2_.

## Results

### Atorvastatin provokes heart defects in zebrafish embryos

**Fig. 1:**
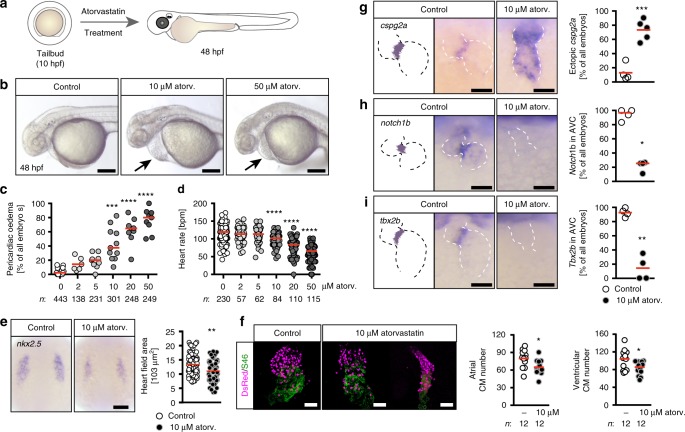
Atorvastatin dose-dependently affects heart formation and function. **a** Zebrafish embryos were treated from tailbud (tb) stage until 48 h post fertilization (hpf) with atorvastatin. **b** Live images of embryos treated either with vehicle (1% DMSO in egg water, Control), 10 or 50 µM atorvastatin. Arrows: pericardiac oedema. Scale bar: 200 µm. **c** Pericardiac oedema formation increases with increasing atorvastatin concentrations; *n* = 16 (0, DMSO), 6 (2 µM), 9 (5 µM), 11 (10 µM), 9 (20 µM), 9 (50 µM); ***p < 0.001, ****p < 0.0001 (atorvastatin versus DMSO control treated embryos), Kruska–Wallis test with Dunn’s multiple comparison test. **d** Heart rate gradually decreases with increasing concentrations of atorvastatin. Each circle is one embryo; *n* = 12 (0, DMSO), 4 (2 µM), 4 (5 µM), 5 (10 µM), 7 (20 µM), 6 (50 µM); ***p < 0.001, ****p < 0.0001 (atorvastatin versus DMSO control treated embryos), Kruskal–Wallis test with Dunn’s multiple comparison test. **e** Atorvastatin affects heart development at the level of cardiac progenitors. In situ hybridization at 8 somite stage (ss) for *nkx2.5*, which labels all cardiac progenitors. Scale bar: 100 µm; *n* = 3 with 72 embryos in total; *p* = 0.0013, two-tailed Mann–Whitney test. **f** Atorvastatin decreases the number of cardiomyocytes. 48 hpf zebrafish hearts expressing DsRed (shown in magenta) in all cardiomyocyte nuclei stained for atrial myosin (S46, green). Scale bar: 50 µm; *p = 0.0167 (atrial cardiomyocytes) and 0.0346 (ventricular cardiomyocytes), two-tailed *t*-test with Welch’s correction. **g** Valve formation is impaired upon atorvastatin treatment. The myocardial gene *cspg2a* is upregulated in atorvastatin embryos at 72 hpf. Red: mean; *n* = 4; *p* = 0.0040, two-tailed, *t*-test with Welch’s correction. **h** Endocardial *notch1B*, which is normally restricted to the atrioventricular canal, is expressed throughout the heart in atorvastatin-treated embryos (72 hpf); *n* = 4; *p = 0.0286, two-tailed, Mann–Whitney test. **i** At 72 hpf, the expression of *tbx2b*, which is normally in the atrioventricular canal, is lost upon atorvastatin treatment. Red: mean; *n* = 4; **p = 0.0014, two-tailed, *t*-test with Welch’s correction. **c**, **f**–**i** Number of embryos given below graphs. **c**–**e**, **g**–**i** Red line indicates median if not otherwise indicated. **g**–**i** Scale bar: 50 µm

We chose zebrafish to investigate a potential impact of statins on cilia function and started with the analysis of heart development in the presence of HMG-CoA-R inhibition. Zebrafish embryos are available in large numbers, develop outside of the maternal womb, can reduce experiments in higher vertebrates such as rodents and can be easily manipulated by addition of pharmacologically active compounds to the culture water. Moreover, zebrafish can be exposed to compounds exclusively during  developmental stages, when cilia form and are functional^17^. Thus, atorvastatin was administered from the onset of organogenesis (tailbud stage), just before the first cilia start to form^18^, until 48 h post fertilization (hpf) (Fig. 1a). The efficacy of atorvastatin to lower cholesterol in zebrafish has been tested previously^19^.

Atorvastatin treatment resulted in embryos with slightly smaller heads and in the formation of pericardiac oedema (Fig. 1b, c), which may be an indicator of impaired heart development. In addition, atorvastatin dose-dependently affected cardiac performance as increasing concentrations of atorvastatin induced bradycardia (Fig. 1d). Co-administration of water-soluble cholesterol (Supplementary Figure 1a) partially rescued the impact of atorvastatin on zebrafish embryos: both the incidence of pericardiac oedema and bradycardia could be significantly reduced (Supplementary Figure 1b-d). Treatment with two other statins, mevastatin and rosuvastatin (Supplementary Figure 2a), caused pericardiac oedema and bradycardia with a similar efficacy as atorvastatin (Supplementary Figure 2b-d), indicating that the observed impact on zebrafish development is not exclusive to atorvastatin. Closer analysis of cardiac morphology revealed that atorvastatin affects heart development already on the level of progenitors. *Nkx2.5*, one of the earliest markers for cardiac progenitor cells, was significantly reduced in the presence of atorvastatin (Fig. 1e). This reduction in cardiac mass persisted even at later stages as atorvastatin-exposed embryos displayed a modest, but reproducible decrease in atrial and ventricular cardiomyocytes compared to dimethyl sulfoxide (DMSO)-treated control embryos (Fig. 1f). This explained why the hearts of atorvastatin-treated embryos looked smaller. In addition, we observed an altered morphology with often elongated hearts (Fig. 1f, panel on the far right). Therefore, we investigated whether atorvastatin treatment interfered with normal development of the atrioventricular canal. Indeed, marker genes for this region were detected throughout the entire heart tube (Fig. 1g) or were completely abrogated (Fig. 1h, i). These results together suggest that statin administration prevents proper heart formation and function in zebrafish embryos.

### Atorvastatin administration results in ciliopathy phenotypes

**Fig. 2:**
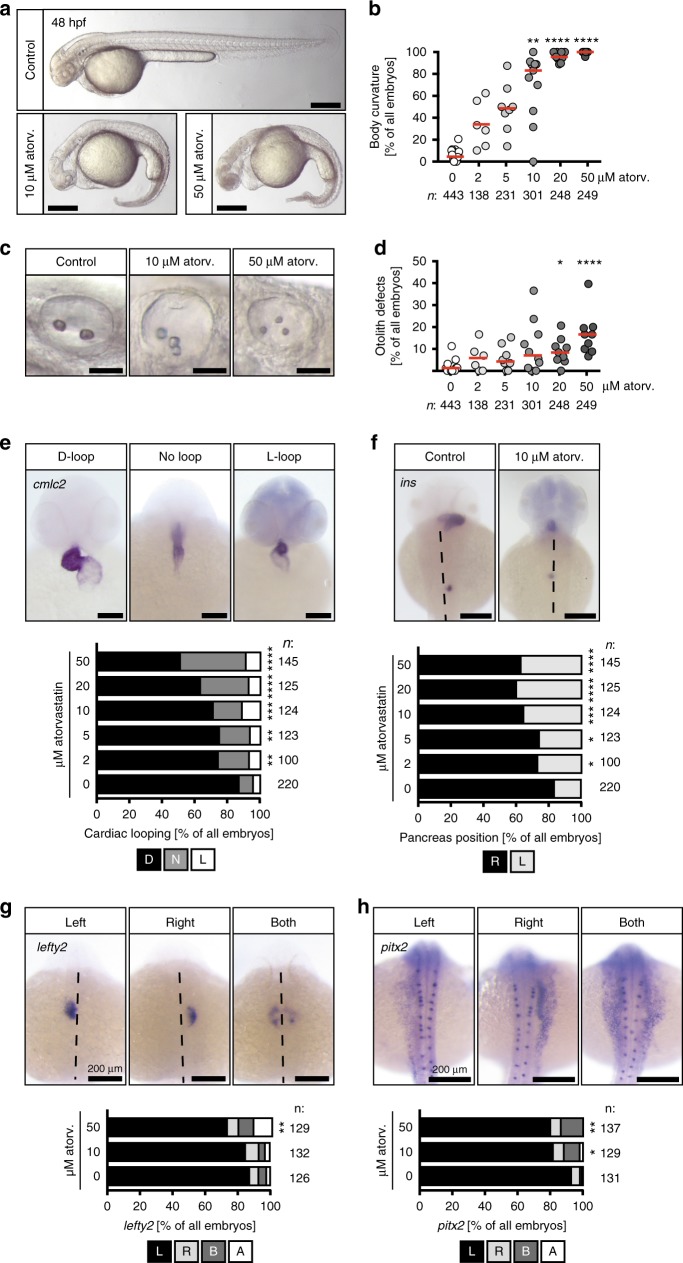
Atorvastatin provokes ciliopathy phenotypes. **a** Atorvastatin treatment results in embryos with curved bodies. Scale bar: 200 µm. **b** Body curvature worsens with increasing atorvastatin concentrations; *n* = 16 (0, DMSO), 6 (2 µM), 8 (5 µM), 11 (10 µM), 9 (20 µM), 9 (50 µM); **p < 0.01, ****p < 0.0001 (atorvastatin versus DMSO control treated embryos), Kruskal–Wallis test with Dunn’s multiple comparison test. **c** Live images of otic placodes at 48 hpf showing defects in otolith formation in atorvastatin-treated embryos. Scale bar: 50 µm. **d** Graph displaying the dose-dependent increase in otolith defects upon atorvastatin treatment; *n* = 15 (0, DMSO), 6 (2 µM), 8 (5 µM), 10 (10 µM), 9 (20 µM), 9 (50 µM); *p < 0.05, ****p < 0.0001 (atorvastatin versus control treated embryos), Kruskal–Wallis test with Dunn’s multiple comparison test. **e** Atorvastatin produces random heart looping. Images of *cmlc2* in situ hybridization to visualize the whole heart at 48 hpf. Scale bar: 100 µm; *n* = 9 (0, DMSO), 6 (2, 5, 10, 20 and 50 µM); *p* = 0.0065 (2 µM), 0.0073 (5 µM), 0.0005 (10 µM), < 0.0001 (20 and 50 µM). **f** Pancreas placement becomes randomized upon atorvastatin treatment. Dorsal views of embryos processed by in situ hybridization for the endocrine pancreas marker *insulin* (*ins*). Dashed line indicates midline of embryo. Scale bar 200 µm. Stacked bar graph summarises 9 (DMSO) and 6 (atorv. concentrations) experiments; *p* = 0.0492 (2 µM), 0.0493 (5 µM), 0.0002 (10 µM), < 0.0001 (20 and 50 µM). **g** The leftward gene *lefty2* is ambiguously expressed after atorvastatin treatment. L expression on left side, R right side, B both sides of midline (dashed), A absent expression. Scale bar: 200 µm; **p = 0.0072. **h** *pitx2* is randomly expressed in the left and/or right lateral plate mesoderm after atorvastatin treatment. L expression on left side, R right side, B both sides of midline (dashed), A absent expression. Scale bar: 200 µm; *p = 0.0155, **p = 0.0071. **b**, **d**–**h** Number of individual embryos given below or next to graphs. **e**–**h** Two-tailed Fisher’s exact tests (atorvastatin versus control treated embryos)

Recently, cilia have emerged as the central organelle controlling heart development^16,20^. Interestingly, atorvastatin as well as mevastatin and rosuvastatin produced zebrafish embryos with a gross morphology reminiscent of cilia defects: embryos exhibited a body curvature, which worsened with increasing concentrations of atorvastatin (Fig. 2a, b, Supplementary Figure 2e). Treatment with statins furthermore disturbed normal otolith seeding, which is controlled by different types of cilia in the otic placode^21^ (Fig. 2c, d, Supplementary Figure 2f). Body curvature and otolith defects of atorvastatin-treated embryos were ameliorated by reconstitution with cholesterol (Supplementary Figure 3). We also observed a thickening of the yolk extension (Fig. 2a), pinheads and smaller eyes, which have been similarly reported for a mutant of HMG-CoA-R^22^ indicating that the ciliopathy phenotypes are likely due to HMG-CoA-R inhibition and no off-target effects. To further validate this finding and investigate the mechanism underlying the heart defect under statins more closely, we assessed left–right (LR) asymmetry development. LR asymmetry is a prerequisite of oriented and faithful heart development. As a consequence, the majority of patients devoid of proper LR asymmetry establishment develop congenital heart defects as heart looping becomes randomized^23^. In addition, other abdominal organs may be erroneously distributed^18,24^. Indeed, zebrafish embryos treated with increasing concentrations of atorvastatin displayed heart looping defects with substantial fractions of all embryos analysed having either inversely looped hearts or such that completely fail to undergo looping (Fig. 2e). Analysis of pancreas placement as detected by in situ hybridization for the endocrine pancreas marker *insulin* revealed further that LR asymmetry was also disturbed on the level of abdominal organs (Fig. 2f). Similarly, early leftward genes such as *lefty2*, which locates to the left cardiac mesoderm, and *pitx2*, which is expressed in the left lateral plate mesoderm, were randomly expressed on both sides of the midline (Fig. 2g, h). The same LR asymmetry defects could be observed upon treatment with other statins, too (Supplementary Figure 4). In contrast to atorvastatin, however, there was an increased incidence of embryos developing a cardia bifida, which had similarly been shown for HMG-CoA-R mutants^22^. Thus, statin treatment produces phenotypes resembling those arising from cilia defects, which includes aberrant cardiac looping.

### Atorvastatin alters LR organizer morphology

**Fig. 3:**
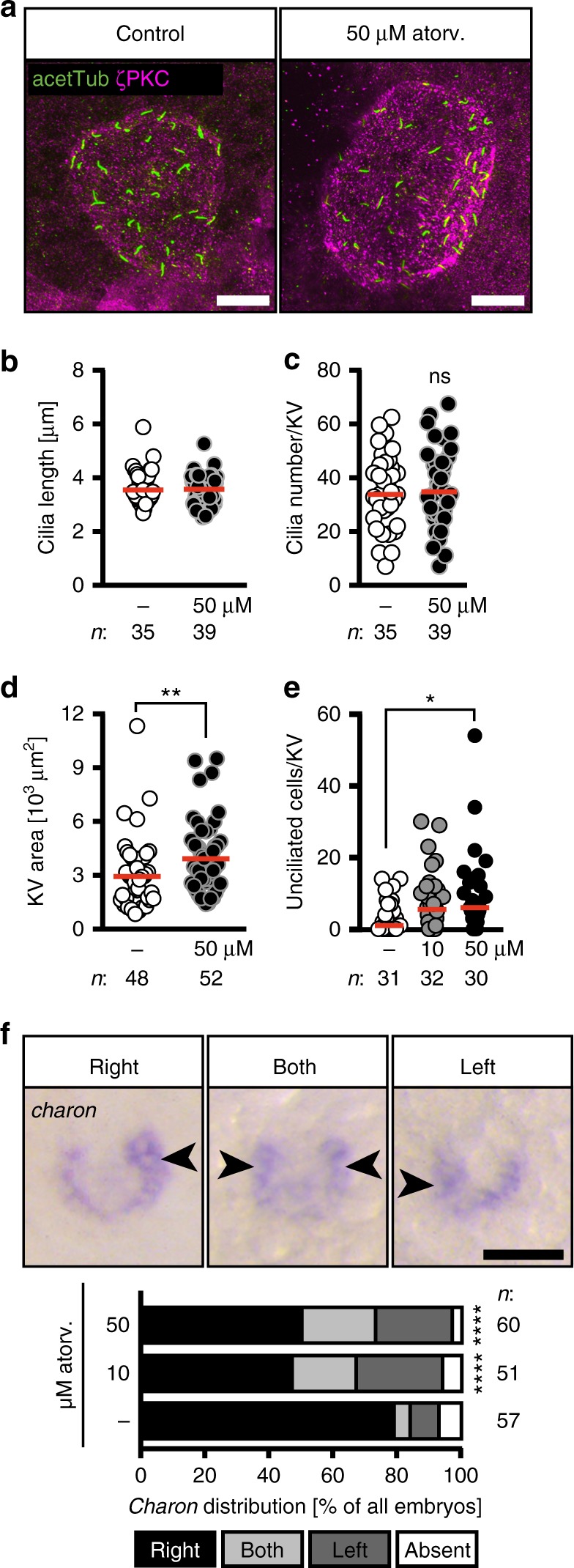
Atorvastatin impacts on the development of the temporal organ of laterality. **a** Confocal stacks of 6–8 somite stage (ss) Kupffer’s vesicle (KV). Cilia were stained with an antibody against acetylated tubulin (acetTub, green). Apical cell membranes to visualize the KV outline were stained with an antibody against atypical PKC (PKCζ, magenta). Scale bar: 20 µm. **b** Bar graph displaying the length of KV cilia in control and atorvastatin-treated embryos; *n* = 1231 (DMSO) and 1424 (50 µM atorv.) cilia; *p* = 0.6357, two-tailed Mann–Whitney test. **c** Cilia number per KV was not changed in the presence of atorvastatin; *p* = 0.6861, two-tailed *t*-test with Welch’s correction. **d** KV area was larger upon atorvastatin treatment. Each circle is one KV and embryo. ** *p*=0.0034. Two-tailed Mann–Whitney test. **e** Atorvastatin-treated embryos have more unciliated cells in the KV than control embryos. Cilia were stained using anti-acetylated antibody and KV cells were counted using the Sox17-GFP line. KV cells express high levels of GFP and can be distinguished from surrounding cells outside the KV. Each circle symbolizes one embryo; *p = 0.0375, Kruskal–Wallis test with Dunn’s multiple comparison test. **f** Atorvastatin disturbs correct spatial expression of *charon*. Representative images of in situ hybridization for *charon* at 10 ss with correct strong expression on right side and aberrant strong expression on both sides or left side (expression indicated by arrowheads; absent expression not shown). Scale bar: 50 µm. Stacked bar graphs summarises three experiments. Fisher’s exact test; *n* = 57 (DMSO), 51 (10 µM atorvastatin) and 60 (50 µM atorvastatin); ****p < 0.0001 for DMSO vs 10 µM as well as vs 50 µM. **b**–**f** Numbers of embryos analysed indicated below or next to graphs

LR asymmetry in zebrafish is determined long before a heart tube develops. It starts with the formation of the Kupffer’s vesicle (KV), the temporal organ of laterality in zebrafish. The KV is a fluid-filled vesicle, which is formed by a monociliated epithelium and is the corresponding structure to the mouse node. Cilia within and at the edge of this KV both propel the vesicular liquid and sense the flow. This results in the asymmetric expression of genes, which at later stages distinguish the left from the right body half and determine the asymmetric placement of inner organs^18,25^. Importantly, the KV ceases to exist after the 18 somite stage when leftward gene expression has robustly been induced^26^. Hence, timed administration of pharmacologically active compounds can be used to block cholesterol synthesis precisely during stages, when the KV and its cilia form and actively determine LR asymmetry. We thus treated zebrafish embryos from tailbud stage to 20 somites with atorvastatin, which coincides with stages, when the ciliated node in the mouse would form and function (embryonic day 7.5–8.5^27^). Treatment during this period was sufficient to generate embryos with the same impairments in cardiac and cilia function (Supplementary Figure 5). Therefore, we concluded that cholesterol may be required for proper functioning of the KV. Neither cilia length nor cilia number per KV, however, were altered upon atorvastatin treatment (Fig. 3a–c). The area of the KV, however, was significantly larger (Fig. 3d). Therefore, we analysed whether all cells of KV extended cilia. We found that atorvastatin increased the number of cells per KV, which failed to form a cilium (Fig. 3e). As this potentially impacts on the flow within the node we assessed expression of the flow sensor *charon*. *Charon* is initially expressed around the whole KV, but becomes downregulated on the left side and asymmetrically expressed when the counter-clockwise, nodal flow is properly established. In the case of irregular or clockwise flow, *Charon* can be found equally expressed on both sides of the KV or more pronounced on the left side^28^. Atorvastatin-treated embryos did in fact show ambiguous expression of *charon* (Fig. 3f), suggesting that disturbed flow may be the underlying cause of the observed LR asymmetry defects. In summary, atorvastatin causes heart defects likely due to a malformation of the temporal organizer of laterality.

### Atorvastatin interferes with cilia formation and signalling

**Fig. 4:**
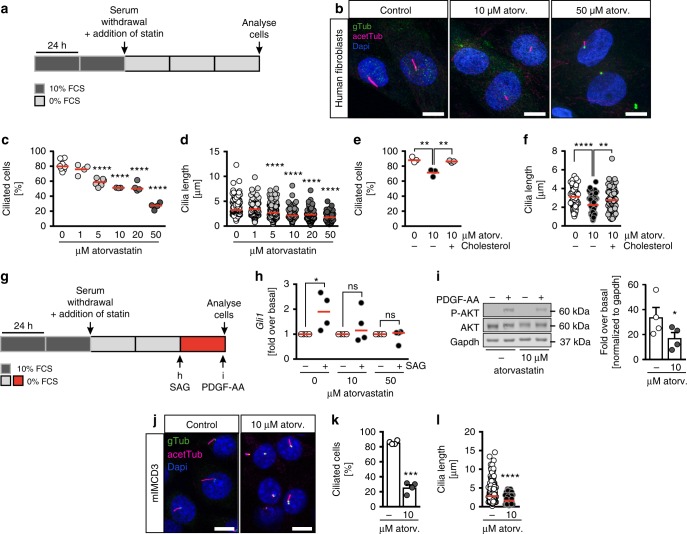
Atorvastatin disrupts primary cilia formation and function in mammalian cells. **a** Cell culture conditions to induce cilium formation by starvation in the presence of atorvastatin. **b** Atorvastatin administration to human fibroblasts results in fewer and shorter cilia compared to DMSO-treated control cells. acetTub (acetylated tubulin, magenta): cilia; gTub (γtubulin, green): basal bodies. Scale bar: 10 µm. **c** Atorvastatin dose-dependently prevents primary cilia formation in serum-starved fibroblasts. Number of cilia/individual treatments: 412/8 (DMSO), 212/4 (1 µM atorv.), 287/6 (5 µM atorv.), 155/3 (10 µM atorv.), 287/4 (20 µM atorv.), 250/4 (50 µM atorv.); ****p < 0.0001 (atorvastatin versus control treated cells). **d** Fibroblast cilia length gradually decreases upon increasing atorvastatin concentrations. Number of cilia/individual treatments: 320/7 (DMSO), 214/4 (1 µM atorv.), 269/6 (5 µM atorv.), 97/4 (10 µM atorv.), 147/4 (20 µM atorv.), 47/2 (50 µM atorv.); ****p = 0.0001. **e** Cholesterol supplementation restores cilia formation in fibroblasts; *n* = 3 treatments with 248–254 cilia; **p = 0.0034 (DMSO vs. 10 µM atorv.), *p* = 0.0073 (10 µM atorv. vs. 10 µM atorv. + cholesterol). **f** Cilia length of human fibroblasts is rescued by cholesterol; *n* = 81–112 cilia from 3 treatments. Circles: individual cilia; **p = 0.0027, ****p < 0.0001. **g** Experimental outline to induce ciliary signalling through SAG or PDGF-AA stimulation. **h** SAG-induced Hedgehog signalling is attenuated by atorvastatin. *p* values (stimulated vs. non-stimulated): 0.0148 (DMSO), 0.6375 (10 µM atorv.), 0.9594 (50 µM atorv.). **i** The response to PDGF-AA stimulation (as measured by AKT phosphorylation at serine 473) is blunted upon atorvastatin treatment compared to DMSO-treated human fibroblasts; *n* = 4. Means ± SEM; *p = 0.0375, two-tailed paired *t*-test after log-transformation of values to obtain normal distribution. **j** mIMCD3 cells cultured as described in (**a**) develop fewer and shorter cilia in the presence of atorvastatin. Representative confocal *z*-stacks; cilia: magenta; basal bodies: green. **k** Atorvastatin results in significantly fewer ciliated mIMCD3 cells. Means ± SEM; *n* = 4. ***p = 0.0002, two-tailed *t*-test with Welch’s correction. **l** Cilia length is reduced under atorvastatin in mIMCD3 cells; *n* = 355 (DMSO) and 188 (atorv.) cilia; ****p < 0.0001, two-tailed Mann–Whitney test. **c**, **e**, **h** One-way ANOVA with Sidak’s post-test. **d**, **f** Kruskal–Wallis test with Dunn’s multiple comparison test

Since residual sterols from the yolk could ameliorate the degree of cholesterol reduction and thus mask a cilia length phenotype in early zebrafish embryos, we analysed cilia also at 24 hpf when more of the yolk had been used up. At this stage, we observed a reduction in cilia length in the neural tube (Supplementary Figure 6) suggesting that atorvastatin does not only interfere with ciliation per se, but potentially with cilium extension, too. In addition, we detected malformations of the pronephric tubes suggesting an additional impact on renal cilia. Embryos treated with atorvastatin developed kidney cysts and showed impaired glomerular fusion and thickening of the distal pronephric tubule (Supplementary Figure 7). Moreover, *Tetrahymena thermophila*, a multiciliated protozoan, had shorter cilia when treated with atorvastatin (Supplementary Figure 8). To investigate atorvastatin’s effects on cilia more closely, we turned to a heterologous cell system, in which lipid abundance can be better controlled than in a whole organism. We used hTert immortalized human fibroblasts and induced primary cilium formation by serum starvation for 72 h with concomitant atorvastatin administration or DMSO application as control (Fig. 4a). Consistent with our observation in the KV of zebrafish embryos, fewer cells extended cilia when atorvastatin was applied during ciliogenesis (Fig. 4b, c). In addition, cilia length gradually decreased with increasing concentrations of atorvastatin (Fig. 4d). To test whether this was due to cholesterol depletion we administered cholesterol to the cells for a short time during starvation. This was sufficient to restore the percentage of ciliated cells as well as the length of cilia (Fig. 4e, f). We next excluded that the shortening of cilia was caused by an overall shrinking of the plasma membrane by measuring the cell size upon atorvastatin treatment. The size of trypsinized fibroblasts was not changed by atorvastatin (Supplementary Figure 9).

Concomitant with the loss of cilia we also detected a failure to induce ciliary signalling. Human fibroblasts were again serum starved and simultaneously treated with atorvastatin. In the last 24 h of starvation, Hedgehog signalling was stimulated using the Smoothened agonist SAG (Fig. 4g). Alternatively, platelet-derived growth factor (PDGF) receptor-α signalling was induced using a short stimulation (3 min) with PDGF-AA (Fig. 4g). While direct stimulation of the Hedgehog signal transducer Smoothened using SAG produced robust target gene expression in control cells, we found target gene transcription completely abrogated in atorvastatin-treated cells (Fig. 4h). This result could also be transferred to zebrafish, which equally displayed reduced Hedgehog signalling upon atorvastatin treatment (Supplementary Figure 10). In addition, we assessed PDGF-AA signalling, which is similarly executed via the primary cilium^29^. PDGF-AA reproducibly triggered AKT phosphorylation in serum-starved control cells, but to a significantly lower extent in atorvastatin-treated fibroblasts (Fig. 4i). To test whether primary cilia of other mammalian cells reacted the same to statin treatment, we treated murine inner medulla and collecting duct (mIMCD3) kidney cells with atorvastatin according to the protocol outlined in Fig. 4a. We saw a robust reduction in ciliation efficiency and shortened cilia in the presence of atorvastatin (Fig. 4j, l). Finally, we assessed the impact of atorvastatin on multiciliated cells. Human airway epithelia cells were cultured at air–liquid interface (ALI) configuration. When atorvastatin was administered to the medium at the day when ALI was established and remained in the medium during the entire differentiation and maturation time, cilia failed to form (Supplementary Figure 11a). In contrast, DMSO-treated control cells had fully polarized as shown by ZO-1 staining and extended the first cilia (Supplementary Figure 11a). When atorvastatin was added on day 14 of ALI culture after ciliation had already started, we did not see changes in the number of ciliated cells, but lesions within the epithelium had formed (Supplementary Figure 11b-d). We hence conclude that atorvastatin treatment disturbs cilium formation and function and that this function likely is conserved from the evolutionarily low one-cell ciliate *Tetrahymena* over vertebrate zebrafish embryos to mammalian cells.

### Atorvastatin-induced defects are likely due to cholesterol depletion

**Fig. 5:**
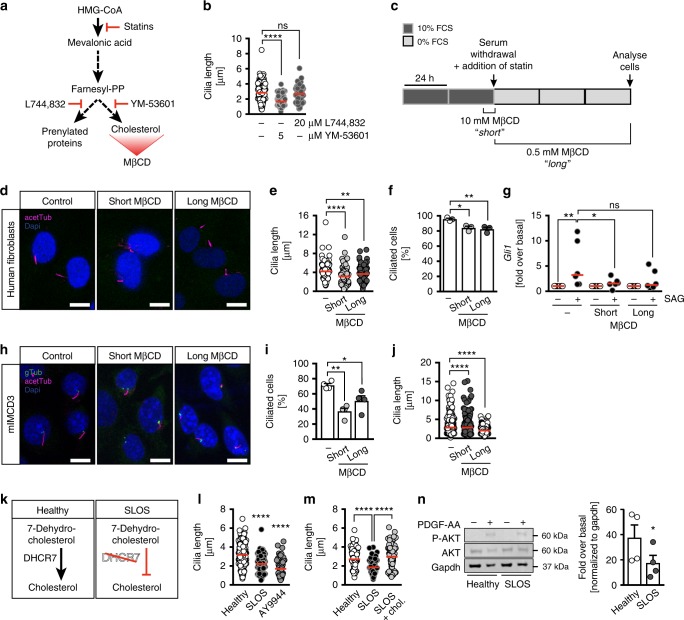
Cholesterol depletion by different means impairs ciliogenesis. **a** Cartoon of cholesterol biosynthesis including administered inhibitors and the cholesterol scavenger methyl-β-cyclodextrin (MβCD). L744,832, farnesyltransferase inhibitor. YM-53601, squalene synthase inhibitor. **b** 5 µM YM-53601 reduces cilia length, 20 µM L744,832 does not; *n* = 75–187 cilia from 4–5 treatments; ****p < 0.0001, ns *p* = 0.4689. **c** Cells were treated either with 10 mM MβCD for a short time during the onset of ciliogenesis or throughout the whole starvation period using 0.5 mM. **d** Confocal *z*-stacks of MβCD-treated human fibroblasts. Scale bar: 10 µm. **e** MβCD treatments result in shorter cilia in fibroblasts; *n* = 128 (DMSO), 108 (short), 135 cilia (long); **p = 0.0026, ****p < 0.0001. **f** MβCD treatment reduces ciliation in fibroblasts. Means ± SEM; *n* = 3; *p = 0.0158, **p = 0.0083. **g** SAG-induced Smoothened signalling is impaired upon MβCD treatment. Cells were treated as depicted in (**c**) and stimulated as in Fig. 4h; *n* = 6; *p = 0.023, **p = 0.0074, ns *p* = 0.0572. **h** Confocal *z*-stacks of mIMCD3 cells treated with MβCD as described in (**c**). Cilia in magenta (acetTub), basal bodies in green (gTub). Scale bar: 10 µm. **i** MβCD treatment reduces ciliation in mIMCD3 cells (same DMSO control as in Fig. 4k); *n* = 4; *p = 0.0265, **p = 0.0013, One-way ANOVA with Dunnett’s multiple comparison test. **j** MβCD shortens cilia in mIMCD3 cells (same DMSO control as in Fig. 4l); *n* = 350 (DMSO), 303 (short) and 275 (long) cilia; ****p < 0.0001. **k** In Smith–Lemli–Opitz syndrome (SLOS) patients, DHCR7 is defective resulting in impaired conversion of 7-dehydrocholesterol to cholesterol. **l** Patient-derived fibroblasts with a genetic variant of *DCH7* (G138V; H405Y) extend shorter cilia than control fibroblasts. Treatment of healthy fibroblasts with 5 µM of the DHCR7 inhibitor AY9944 shortens cilia; *n* = 282 (DMSO), 161 (SLOS) and 65 cilia (AY9944); ****p < 0.0001 (compared to DMSO). **m** Cilia length of SLOS patient cells is restored after cholesterol supplementation; *n* = 64–77 cilia; ****p < 0.0001. **n** Blunted response to PDGF-AA in SLOS fibroblasts; *n* = 4. Means ± SEM; *p = 0.0201, two-tailed paired *t*-test after log-transformation of values to achieve normal distribution. **b**, **e**, **j**, **l**, **m** Kruskal–Wallis test with Dunn’s multiple comparison test. **f**, **g** One-way ANOVA with Holm–Sidak’s multiple comparison test

Cholesterol is one of several end-products of the mevalonate pathway, which is inhibited by statins at a very early step^30^ (Fig. 5a). To test whether the observed effects originated from a loss in cellular cholesterol, we applied specific inhibitors, which block either farnesyltransferase, the enzyme catalysing the formation of prenylated proteins, or squalene synthase that is required for the generation of cholesterol. In our hands, only inhibition of squalene synthase via YM-53601 treatment significantly reduced cilia length in human fibroblasts (Fig. 5b). We further corroborated this finding by depleting human fibroblasts from free cholesterol using the scavenger methyl-β-cyclodextrin (MβCD) (Fig. 5c). MβCD is commonly used to efficiently deplete cells acutely from free cholesterol^31^. Both, cholesterol depletion by MβCD right before inducing ciliogenesis by starvation and persistent cholesterol depletion during starvation resulted in shorter and fewer cilia (Fig. 5d–f), indicating that cholesterol is not only required to modify Hedgehog signalling molecules^32^, but also to allow for correct ciliogenesis. Both MβCD treatment regiments also abrogated Hedgehog signalling in human fibroblasts (Fig. 5g) and decreased the percentage of ciliated cells and the length of cilia in mIMCD3 cells (Fig. 5h–j). Finally, we measured cilia length in cells derived from a SLOS patient who has been identified as a compound heterozygote harbouring a G to T transversion in the *DHCR7* gene in one allele (c.413G>T, resulting in an amino acid change: Gly138Val) and a C>T transition at nucleotide 1213 (c.1213C>T) resulting in the substitution of tyrosine for histidine at position 405 (His405Tyr). Besides partial syndactyly this patient had been diagnosed with failure to thrive, developmental delay, hypotonia, microcephaly and micrognathia. Fibroblasts of this patient produce less cholesterol and display an accumulation of 7-dehydrocholesterol as well as oxidized products thereof^33^. We found that cilia in cells of this patient are significantly shorter than in control cells (Fig. 5k–m), which could be mimicked by pharmacological inhibition of 7-dehydrocholesterol reductase, the enzyme defective in the SLOS cells used in here^34^, using the compound AY9944 (Fig. 5l). Addition of external cholesterol restored cilium length in SLOS patient fibroblasts (Fig. 5m). Last, but not least, just like in atorvastatin-treated cells, PDGF-AA signalling was blunted in SLOS cells compared to healthy cells (Fig. 5n). These results strongly indicate that cholesterol is required to allow for cilium formation and function.

### Cholesterol depletion alters ciliary phosphoinositide levels

**Fig. 6:**
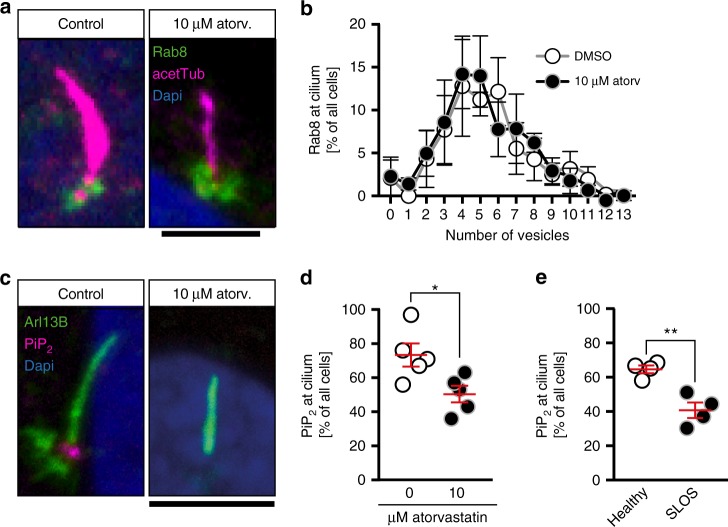
Cholesterol depletion alters phosphoinositide levels at the base of the cilium. **a** Confocal stacks of cilia showing Rab8-positive vesicles at the base of the cilium of human fibroblasts. Cilia were stained with an antibody against acetylated tubulin (acetTub, magenta). Vesicles are labelled in green (Rab8). Scale bar: 4 µm. **b** Distribution of Rab8 vesicle content at the base of the cilium between control treated and atorvastatin-treated human fibroblasts. Means ± SEM; *n* = 3 independent experiments. **c** Confocal stacks showing lack of Pi(4,5)P_2_ (magenta) at the base of cilia, when cells were treated with atorvastatin during ciliogenesis. Cilia were labelled with an antibody against Arl13B (green). Scale bar: 4 µm. **d** Analysis of five independent experiments revealed a significant reduction of cells exhibiting Pi(4,5)P_2_ at the base of the cilium. Each circle indicates the percentage of cells having Pi(4,5)P_2_ at the base of the cilium in one experiment. Means ± SEM; *n* = 5 with 181–202 cilia in total; *p = 0.0269, two-tailed *t*-test with Welch’s correction. **e** SLOS patient-derived fibroblasts exhibit less Pi(4,5)P_2_ at the base of the cilium. Means ± SEM; *n* = 4 with 154–156 cilia in total; **p = 0.0071, two-tailed *t*-test with Welch’s correction

In order to uncover how cholesterol facilitates and statins hence interfere with cilium extension, we examined Rab8 localization in fibroblasts. Rab8 is a small Rab GTPase involved in the vesicular transport from the trans-Golgi network to the cilium. Importantly, it facilitates not only the transport of receptors towards the cilium, but also membrane components to elongate the ciliary membrane^35–37^. Thus, the observed reduction in cilium length could be due to a deficiency in vesicular transport mechanisms. Analysis of Rab8 vesicles in human fibroblasts revealed in fact a slightly reduced number of Rab8-positive vesicles at the base of cilia upon atorvastatin treatment (vesicle number_DMSO_: 4.794 ± 0.1646 (107 cilia), vesicle number_10 µM atorv._: 4.286 ± 0.1471 (112 cilia); *p* = 0.0222, unpaired *t*-test with Welch’s correction). However, we did not consider a defect in Rab8-mediated transport as a likely cause for the observed cilia phenotypes as the overall distribution of Rab vesicles at the base of the cilium was not altered (Fig. 6a, b).

We further investigated whether Pi(4,5)P_2_ correctly localised to the base of the cilium. Pi(4,5)P_2_ is a phosphoinositide, which likely functions as an entrance guide at the base of the cilium^14,38^. Disruption of its transition zone localisation could explain the ciliogenesis defects we observe. Moreover, Pi(4,5)P_2_ is formed from Pi(4)P by phosphorylation and the synthesis of Pi(4)P in turn depends on membrane sterol levels at the Golgi^39–41^. We therefore analysed whether Pi(4,5)P_2_ localised correctly to the ciliary base using immunofluorescence. Atorvastatin significantly reduced the number of cilia with Pi(4,5)P_2_ (Fig. 6c, d). Similarly, cilia of SLOS cells had less often Pi(4,5)P_2_ at the cilium (Fig. 6e). Thus, cholesterol depletion by atorvastatin is likely to disrupt proper ciliogenesis by altering phosphoinositide levels at the cilium.

## Discussion

The developing foetus relies on endogenous as well as exogenous cholesterol^2^. This knowledge is largely based on studies in model organisms in which HMG-CoA-R inhibitors have been reported to induce anomalies of different degrees during embryonic development^42,43^. So far, however, developmental defects caused by statins have mostly been attributed to defects in prenylation rather than cholesterol synthesis^22,44^. This is surprising because cholesterol is equally required for foetal development as prenylation. Moreover, patients suffering from insufficient cholesterol synthesis develop congenital malformations^2,6^. Since such cholesterol depletion malformations resemble those caused by cilia defects, we tested here whether cholesterol abrogation by statins or other means induces cilia defects.

Up to now it has been known that cholesterol modulates Hedgehog signalling and hence cilia function through modification of different pathway components^32,45-49^. Using zebrafish as well as human fibroblasts we here provide evidence that statins and other ways of cholesterol depletion cause not only cilium dysfunction, but also affect formation of the cilium. In zebrafish, we observed malformations of the head, ear, kidney, body curvature as well as a pronounced impairment of cardiac performance and morphogenesis. Reduced head circumference and heart defects are malformations, which also occur frequently in SLOS patients^6^ and by now it is well established that cilia dysfunction can contribute to the development of both microcephaly and congenital heart defects^7,50^. Consistently, the phenotypes we observed in zebrafish were reminiscent of general cilia dysfunction. This included disruption of LR asymmetry development, which is a prerequisite for oriented organ development. LR symmetry breaking is further indispensable for proper cardiogenesis as a large fraction of patients with situs anomalies suffer from structural heart defects^24,50^. Closer analysis revealed further that besides functionality, formation of the cilium is hindered, too, when zebrafish embryos are exposed to statins.

In human fibroblasts and murine IMCD3 cells, cholesterol depletion produced similar defects in cilium formation as observed in zebrafish. Moreover, human airway epithelial cells failed to form a continuous, ciliated epithelial cell layer when atorvastatin was applied on the first day of ALI culture. This suggests that our data obtained in the low vertebrate zebrafish can likely be transferred to higher vertebrates and potentially even patients. The observed defect in cilium extension was further accompanied by a loss of canonical Hedgehog signalling and an impairment of PDGF-AA signalling. Lipid disorders such as SLOS have always been linked to an attenuation in Hedgehog signalling^9,32,45^. Hedgehog as well as its signal transducer Smoothened require cholesterol modifications for full activity^51^. Moreover, cholesterol and derivatives thereof bind to Smoothened and activate Smoothened-mediated signal transduction^46–48,52^. Therefore, loss of cholesterol by any means abrogates Hedgehog signalling and produces malformations of organs, whose development is governed by the Hedgehog cascade. The Hedgehog cascade, however, is also contingent on the structural integrity of primary cilia^53^, suggesting that cholesterol-mediated alterations in the structure of cilia (including the length) are upstream of any effects on cilia function. Indeed, changes in cilia length for instance directly correlate with the level of Hedgehog activity^54,55^. Alterations in cilia may thus contribute or be upstream of Hedgehog signalling attenuation when cholesterol is reduced. Although diminished Hedgehog signalling impacts on cilia formation, too, as zebrafish mutants for Smoothened develop shorter cilia in their KVs^56^, the reduction in cilia length seems not pronounced enough to postulate Hedgehog signalling as the major determinant of cilia length and thus an upstream regulator of cilium formation in SLOS. Therefore, we propose that sufficient amounts of cholesterol are required for faithful ciliogenesis, which in turn facilitates and propagates ciliary signalling and hence allows for correct embryonic development.

The molecular mechanism of how cholesterol executes this function is potentially based on cholesterol being a membrane component. Membrane extension of the growing cilium requires continuing vesicular trafficking to the base of the cilium, which contains no ribosomes or biosynthesis machinery^57^. Cholesterol is not only enriched in certain vesicles^58^, but also vesicle docking as well as trafficking rely on cholesterol^59^. Moreover, membrane sterol levels in the trans-Golgi network positively modulate the synthesis of the phosphoinositide Pi(4)P^39^ by enhancing the activity of Pi4 kinases^39,41^. Since Pi4 kinases potentially also regulate vesicular flux^60^, failure in proper cilia extension in SLOS patients could be a defect in vesicular transport. We thus examined Rab8-positive endosomes because Rab8-mediated vesicular trafficking had been shown crucial for proper ciliogenesis: inhibition of Rab8 by expressing a dominant negative construct of Rab8 prevents cilium formation in cell culture and causes KV abnormalities in zebrafish^37^. In principle, this was consistent with our findings of morphological changes in the KV of atorvastatin-treated zebrafish. However, we neither detected Rab8 along the cilium as previously reported^37^ nor did we observe large changes in vesicle accumulation at the base of the cilium. Therefore, we dismissed the hypothesis that trafficking to the cilium is deficient and causative for the ciliogenesis defects observed upon cholesterol depletion.

**Fig. 7:**
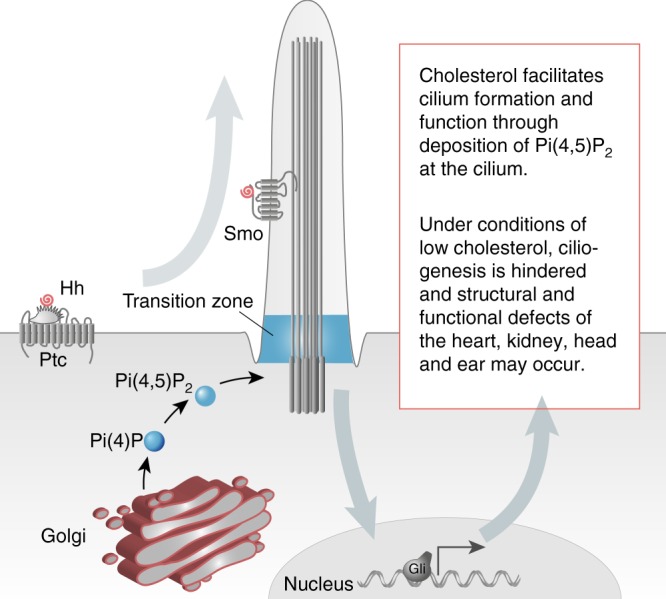
Proposed model of cholesterol function in ciliogenesis. Cholesterol in the trans-Golgi network is required for the synthesis of Pi(4)P, which in turn is phosphorylated to Pi(4,5)P_2._ Pi(4,5)P_2_ is an integral component of the transition zone of the cilium and hence controls the import and potentially also the export of cargo to and from cilia. This facilitates ciliary signalling such as Hedgehog signalling, which in turn is required for proper development of the heart and anterior structures such as the head and the ear. The Hedgehog cascade is further propagated by cholesterol modifications of Hedgehog (Hh, bound to Patched, Ptc) and of its signal transducer Smoothened (Smo). In the absence of cholesterol, Pi(4,5)P_2_ accumulation at the cilium is inhibited resulting in impaired cilium formation. In addition, ciliary signalling, for instance Hedgehog signalling, is defective giving rise to ciliopathy phenotypes, which overlap with some of the clinical manifestations of SLOS (i.e., CHD and kidney defects)

Cilium growth, however, not only relies on supplementation from the cell body. Any cargo delivered to the cilium is scrutinized to ensure that only ciliary components enter the cilium. The transition zone at the base of the cilium functions as such a gatekeeper and excludes factors not belonging into the cilium from entering. It further serves as a recruitment platform for proteins indispensable for structural and functional integrity of the cilium^61^. Phosphoinositides, which are enriched in membranes, have been reported to be essential for the establishment of the transition zone. Here, in particular Pi(4,5)P_2_ is important. When Pi(4,5)P_2_ resides not only in the membrane at the level of the transition zone, but leaks further into the cilium, ciliary Hedgehog signalling is inhibited^14^. In our hands, however, we never observed Pi(4,5)P_2_ along the ciliary shaft. Instead we found that a substantial fraction of cells, which still extended a cilium, lost Pi(4,5)P_2_ at the cilium. Interestingly, abrogation of Pi(4,5)P_2_ in *Drosophila* disrupts sperm tail formation and interferes with the initial steps of ciliogenesis by regulating microtubule organization and axoneme extension^38^. We hence propose a model in which cholesterol controls trafficking into and out of cilia by altering phosphoinositide levels and distribution (Fig. 7). Cholesterol depletion, for instance by statin treatment, hence impairs ciliogenesis by disrupting synthesis and transition zone localization of Pi(4,5)P_2_. Thus, despite the retrospective data providing no causal association between statin use in the first trimester and congenital malformations^3,5^, our results nevertheless advise caution towards statin medication during pregnancy.

## Materials and methods

### Materials

Atorvastatin and L744,832 were purchased from Santa Cruz Biotechnology Inc., Germany. Mevastatin, methyl-β-cyclodextrin and water-soluble cholesterol were obtained from Sigma-Aldrich, Germany. YM-53601 and SAG (smoothened agonist) were from Cayman Chemical, USA. AY9944 was from Tocris Bioscience, UK. DNA oligonucleotides were purchased from IDT (Leuven, Belgium). Pravastatin and rosuvastatin were bought from Selleckchem (via Absource Diagnostics, Munich, Germany). Recombinant PDGF-AA was purchased from BioLegend, USA. Human hTert immortalized 1BR3 wild-type fibroblasts were kindly provided by Professor Dr. Penelope A. Jeggo of the Genome Damage and Stability Centre, University of Sussex, UK. The following cell line was obtained from the NIGMS Human Genetic Cell Repository at the Coriell Institute for Medical Research: GM05788. Mouse inner medullar collecting duct cells (mIMCD3) were kindly provided by Sören Lienkamp, Freiburg, Germany. Human bronchial epithelial cell and culture media as well as supplements were purchased from PromoCell (Heidelberg, Germany). *Tetrahymena* (strain CU428) were a gift of Dr. Dorota Wloga at the Nencki Institut, Warsaw, Poland. Primary antibodies used in this project were rabbit anti-AKT (1:1000, clone C67E7, lot no. 17, Cell Signaling, USA), mouse anti-phosphoAKT (1:1000, cat. no. 200–301268S, lot no. 27843, Rockland, USA), rabbit anti-Arl13b (1:300, cat. no. 17711-1-AP, lot no. 00049885, Proteintech, UK), mouse anti-Gapdh (1:500, cat. no. MAB374, lot. no. NG1722294, Millipore, Germany), mouse anti-PiP_2_ (1:100, clone PIP2 2C11, lot no. CRT/14/52, Novus Biologicals, Germany), rabbit anti-Rab8 (1:200, clone D22D8, lot no. 2, Cell Signaling Technologies, Germany), mouse anti-acetylated tubulin (1:500, clone 6-11B-1, lot no. 034M4828, Sigma-Aldrich, Germany), rabbit anti-γTubulin (1:500, cat. no. T5192, lot no. 109K4802, Sigma-Aldrich, Germany), rabbit anti-Ds-Red (1:500, cat. no. 632496, lot. no. 8040464, Clontech, USA), rabbit anti-ZO-1 (1:500, cat. no. 61-7300, lot no. QG215365, Thermo Fisher, Germany), rabbit anti-PKCζ (1:500, cat. no. sc-216, lot no. G2211, Santa Cruz, USA) and mouse anti-S46 (DSHB Hybridoma Product S46, no lot number available). Secondary antibodies were Alexa-coupled 488 or 568 (1:1000, Molecular Probes, Germany) or IR-Dye-coupled for western blot detection (Li-COR, Germany).

### Zebrafish husbandry and manipulation of fertilized eggs

Zebrafish were kept in a circulating water system at 27.5–29 °C with a 14 h day and 10 h night cycle. Fish were fed three times per day. Fertilized eggs were obtained by natural mating of adult zebrafish (4–24 months of age) and incubated at 28.5 °C until the desired stage. Dead or unfertilized eggs were sorted out. Zebrafish lines used in this study were AB wild-types, cmlc2-nucDsRed^62^, Wt1b-GFP^63^ and Sox17-GFP^64^. Drug treatments were performed in 24-well plates in 1 ml egg water supplemented with 0.003% phenyl-thiourea and 1% DMSO as vehicle or drugs dissolved in DMSO as indicated from tailbud stage on. The size of experimental groups depended on the number of fertilized eggs per clutch. Clutches were randomly and equally divided into treatment groups and raised until further analysed. Otherwise no statistical methods have been applied to pre-determine sample size. As there are always embryos also in control groups, which do not survive, the size of the finally analysed groups could not be held equal. All zebrafish experiments were done at least three times, each time with groups of at least 10 embryos and analysed, whenever possible, in a blinded fashion. The numbers of the individual embryos analysed are given within the graphs. Maintenance and manipulation of zebrafish were in compliance with ethical regulations and approved by the Veterinary Care Unit at Ulm University (“Tierschutzbeauftragte”) as well as the animal welfare commissioner of the regional board for scientific animal experiments in Tübingen, Germany. Experiments were performed according to the European Union Directive 86/609/EEC for the protection of animals, which are intended for experimental and other scientific purposes.

### Zebrafish morphology, heart function and kidney formation

Live zebrafish were analysed at the 48 hpf stage and examined for body curvature, pericardiac oedema and otolith defects. In addition, heart rates were counted using an upright microscope with brightfield illumination (see “Imaging”). In detail, zebrafish embryos were placed into a Petri dish with pre-warmed embryo water (28 °C) and the number of heart beats was manually counted for 10 s. Then, beats per minute were calculated. Kidney formation was assessed with the help of the Wt1b-GFP transgenic line at 48 and 72 hpf.

### Analysis of cardiomyocyte number in zebrafish

Zebrafish expressing DsRed under the control of the cmlc2 promotor^62^ were treated with atorvastatin or DMSO from tailbud stage on and fixed at 48 hpf with 1% formaldehyde for 50 min at room temperature (RT). After washing with PBS, embryos were incubated for 1 h in blocking solution containing 10% normal goat serum (Vectorlabs, UK), 2 mg/ml bovine serum albumin (BSA) and 0.2% saponin in phosphate-buffered saline (PBS). Primary antibodies diluted in PBS with 0.2% saponin were added to the embryos and incubated overnight at 4 °C. An antibody against DsRed was used to enhance the fluorescence of the DsRed. Atrial myosin was stained with the S46 antibody to distinguish atrial from ventricular cardiomyocytes. Next day, samples were washed several times for 15 min with PBS supplemented with 0.2% saponin and subsequently incubated in secondary antibodies (diluted in blocking solution) for 3 h at RT, followed by several washing steps with PBS. After mounting the embryos with Vectastain containing 4′,6-diamidino-2-phenylindole (DAPI; Vectorlabs) between coverslips, samples were stored at 4 °C until imaging via confocal microscopy.

### In situ hybridization

Whole mount in situ hybridization was performed according to standard protocols. The SP6 or T7 polymerase (NEB, Germany) along with the DIG RNA labelling system (Roche, Germany) were used to in vitro transcribe probes from linearised plasmids. To detect *charon* transcripts a 583 bp fragment of the complementary DNA (cDNA; NM_212969) was cloned by TOPO TA cloning into pCRII, from which a DIG-labelled probe was transcribed after linearization. All other probes used in this project have been described before: *cardiac myosin light chain 2* (*cmlc2*), *chondroitin sulphate proteoglycane 2a (cspg2a)*, *insulin* (*ins*), *lefty2*, *notch1b, nkx2.5*, *pitx2*^65^ and *tbx2b*^66,67^.

### Cell culture

Human hTert immortalized 1BR3 wild-type fibroblasts (Genome Damage and Stability Centre, University of Sussex, UK) were cultured in minimum essential medium (MEM) Alpha supplemented with 1% penicillin/streptomycin (Pen/Strep) and 10% foetal calf serum (FCS, all from Life Technologies, Germany) at 37 °C in a humidified atmosphere containing 5% CO_2_. Primary SLOS patient fibroblasts (clone GM05788) from a compound heterozygous patient with mutations in *7DHC* reductase were obtained from the NIGMS Human Genetic Cell Repository at the Coriell Institute for Medical Research. As control, non-immortalised 1BR3 cells (Genome Damage and Stability Centre, University of Sussex, UK) were used. Primary cells were cultured in the presence of 15% FCS. mIMCD3 were cultured in Dulbecco's modified Eagle's medium (DMEM):F12 medium completed with 1% Pen/Strep and 10% FCS. All cells have been regularly checked for mycoplasma contamination and have been tested free of mycoplasma. The day before starting an experiment, 150,000 cells/well were seeded in a 6-well plate. On the following day, cells were changed from full to starvation medium (MEM Alpha or DMEM:F12 with 1% Pen/Strep and 0.1% FCS) supplemented with the pharmacologically active compounds or DMSO for 72 h. To assess Hedgehog pathway activity, serum-starved cells were treated with 500 nM SAG during the last 24 h of starvation. To monitor PDGF pathway activity, cells were stimulated using 50 nM PDGF-AA for 3 min at the end of the 3 days of starvation.

### ALI cultures

ALI cultures were essentially performed as previously described^68^. In detail, cryo-preserved primary human bronchial epithelial cells from healthy donors (Epithelix, EP51AB, Genève Switzerland) were thawn and suspended in 75 cm^2^ culture flasks with 20 ml airway epithelial cell basal medium supplemented with airway epithelial cell growth medium supplement pack (catalogue nos. C-21260 and C-39160, PromoCell, Germany) at 37 °C, 5% CO_2_ and 95% humidity. Then, 10 ml of the medium was changed every second day until 90% confluency was reached. Cells were detached using the DetachKit of Promocell (cat. no. C-41200). To establish ALI epithelia, 30–40 × 10^3^ hTEpC were seeded onto collagen-coated (cat. no. 04902, StemCell Technology, Germany) transwell filters (cat. no. 3470, Thermo Fisher). Filters were placed in 24-well plates and wells were filled with 600 µl each of airway epithelial cell basal medium supplemented containing airway epithelial cell growth medium supplement pack. Cells were cultivated on filters under submerged conditions until confluence was reached (usually for 4 days) at 37 °C, 5% CO_2_ and 95% humidity. Then, apical medium was aspirated and basolateral medium was replaced by ALI medium containing DMEM-H and LHC basal medium (both from Gibco via Thermo Fisher, Germany) at a 1:1 ratio and was supplemented with: 0.87 µM insulin, 0.21 µM hydrocortisone, 0.5 ng/ml human epidermal growth factor, 0.01 µM triiodothyronine, 0.125 µM transferrin, 2.7 µM epinephrine, 10 µg/ml bovine pituitary extract, 0.5 mg/ml bovine serum albumin (all from Promocell, Germany), 0.5 µM phosphorylethanolamine, 0.5 µM ethanolamine, 3 µM zinc sulphate, 0.05 µM retinoic acid, 1.5 µM ferrous sulphate, 0.11 mM calcium chloride, 0.6 mM magnesium chloride, 30 µM sodium selenite, 1 µM manganese chloride, 0.5 nM sodium silicate, 1 µM ammonium molybdate tetrahydrate, 5 µM ammonium metavanadate, 1 µM nickel sulphate, 0.5 µM tin chloride (all from Sigma-Aldrich GmbH, Germany), 100 U/ml penicillin and 100 µg/ml streptomycin (both from Gibco via Thermo Fisher, Germany). Basolateral medium was changed every second day. From day 14 on, additional PBS washing steps were performed two times per week to avoid mucus accumulation. To address the impact of atorvastatin on ciliogenesis, cells were either treated from day 1 of epithelial differentiation by addition of atorvastatin to the basolateral medium and fixed on day 14 or treated from day 14 until day 28. Cells were then fixed with 4% paraformaldehyde (PFA) followed by a second fixation with MeOH.

### MβCD assay

Two different approaches were used to deplete cholesterol from 1BR3 fibroblasts or mIMCD3 cells with MβCD. Cells were treated with 10 mM MβCD for 30 min prior to starvation or incubated in 0.5 mM MβCD during the whole course of starvation. In brief, cells cultured in full medium were washed once with PBS and changed to serum free medium supplemented with MβCD to a final concentration of 10 mM. After 30 min of incubation, cells were washed again with PBS and changed to serum starvation medium. For long-term treatments, MβCD was added to cells changed to starvation medium at a concentration of 0.5 mM and incubated until fixation.

### Cholesterol replenishment

For rescue experiments, water-soluble cholesterol was used and titrated to the maximal concentration, which did not induce cell death. SLOS patient and control cells were incubated with 5 mM water-soluble cholesterol 1 h prior to starvation. After washing with PBS, cells were starved for 72 h.

As additional cholesterol proved highly toxic to healthy control cells, a different treatment strategy was used for 1BR3 cells treated with atorvastatin: at the onset of starvation, 10 µM atorvastatin was added. After 48 h, replenishment was performed by co-treatment with 5 mM cholesterol for 1 h. Afterwards, starvation and statin treatment was continued for another 24 h until fixation. In zebrafish, 10 µM water-soluble cholesterol was added along with 10 µM atorvastatin from tailbud until the 48 hpf stage.

### Immunofluorescence

Zebrafish embryos were treated from tailbud stage until fixation at either 6–8 somite stage for KV analyses or at 24 hpf for neural tube analysis. Fixation was done at 4 °C overnight using 4% buffered PFA. Permeabilization and immunofluorescence staining of zebrafish embryos were performed as previously published^69^.

Cells grown on coverslips were fixed with 100% chilled methanol for 5 min or 4% PFA at room temperature for 10 min. ALI cultures were fixed using 4% PFA followed by a second fixation with MeOH. After fixation, cells and filters were washed with PBS, permeabilized for 10 min (cells) or 15 min (filters) with 1% Triton X-100 in PBS, blocked for at least 1 h in PBS supplemented with 10% normal goat serum (Vectorlabs, UK) and incubated in primary antibody diluted in blocking buffer. Next morning, the samples were washed three times for 10 min using PBS and incubated at room temperature in the dark with the secondary antibody diluted in blocking buffer (1:1000). Finally, coverslips and ALI filters were mounted with Vectastain containing DAPI (Vectorlabs, UK) and sealed with nail polish. For cilia length analysis in human fibroblasts and mIMCD3 cells, at least 30 cilia per treatment and experiment were imaged. To assess the percentage of ciliated cells, at least 100 cells per treatment condition and experiment were counted. Each experiment was performed at least 3 times.

### Analysis of area and cell number of the KV

In confocal stacks, protein kinase C ζ (PKCζ) staining was used to visualize the outline of the KV, which was then traced with ImageJ to calculate the KV area. The number of cells per KV was counted in Sox17-GFP embryos, in which brightly fluorescing cells represent KV cells.

### PDGF assay and western blotting

1BR3 wild-type or SLOS patient cells seeded in 12-well plates were serum starved for 3 days and the wild-type cells in addition treated with 10 µM atorvastatin. Before starting lysis in 60 µl cell lysis buffer (2% SDS, 1× phosphatase and protease inhibitors (both Roche, Germany), 50 mM Tris pH 6.8), the cells were stimulated for 3 min with 50 ng/ml PDGF-AA. DMSO supplementation was used as vehicle control for all treatments. Lysates were supplemented with 0.5 µl Pierce universal nuclease (Thermo Fisher), cleared by centrifugation and protein content was analysed with the Bicinchoninic acid (BCA) assay kit (Sigma-Aldrich, Germany). Equal amounts of proteins were separated on a Bolt Bis-Tris Mini gradient gel (4–12 %) in a Bolt Mini Gel tank filled with MES SDS running buffer (all from Life Technologies, Germany) and blotted at 25 V for 1 h onto a nitrocellulose membrane (Bio-Rad Laboratories, Germany) in a X Cell II blot module (Life Technologies, USA). Blocking of the membrane was performed with 3% milk powder in Tris-buffered saline containing 0.1% Tween-20 (TBST) for 1 h at RT followed by overnight incubation at 4 °C with primary antibodies diluted in TBST containing 3% BSA. Next day, blots were washed three times in TBST containing 0.2% BSA and 0.2% NP-40, respectively, and incubated for 1 h at RT in IR-Dye-coupled secondary antibodies. Western blot signals were detected, normalized and quantified with a LI-COR Odyssey SA system and software. Antibodies used for pathway analyses were anti-AKT and anti-phospho-AKT, whereas glyceraldehyde 3-phosphate dehydrogenase (GAPDH) served as loading control.

### qPCR

Total RNA was isolated from fibroblasts or zebrafish embryos using the Quick-RNA MiniPrep kit (Zymo, USA). cDNA was synthesized by Superscript III reverse transcriptase with oligodTTPs (Life Technologies, Germany) using equal amounts of total RNA as template. qPCR was run in duplicates or triplicates on a LightCycler 480 with the Universal Probe System (both Roche, Germany) and the GoTaq Probe qPCR Master Mix (Promega, USA). The following primers and probes were used: human *SDHA*: forward (Fw): 5′-GGA CCT GGT TGT CTT TGG TC, reverse (Rev): 5′-CCA GCG TTT GGT TTA ATT GG, Universal Probe 80; human *GLI1*: Fw: 5′-CCA GCC AGA GAG ACC AAC AG, Rev: 5′-CCC GCT TCT TGG TCA ACT T, Universal Probe 7; zebrafish *b2m*: Fw: 5′-ACA TCA CTG TAC AGG GGA AAG TC, Rev: 5′-TCC GTT CTT CAG CAG TTC AA, Universal probe 65; zebrafish *gli1*: Fw: 5′-GGT CTC GAT GCC AGT GGA, Rev: 5′-CAC TGA CGG AGC CAG TCC, Universal probe 5.

### Imaging

For cilia analyses *z*-stacks were acquired using a Leica TCS SP5II confocal microscope with a section distance of 0.3 µm. The stacks were merged using the 3D projection tool in the Leica acquisition software of the confocal and imported into ImageJ^70^. With the help of a Wacom tablet individual cilia were traced and the length of the trace was measured in ImageJ. In *Tetrahymena*, only cilia in the central portion of the cell were measured. A Zeiss Axiophot epifluorescence microscope was used to verify and analyse staining efficiency and to image ALI cultures. All zebrafish live or whole mount in situ hybridization images were acquired with a Leica M125 upright microscope and a Leica IC80 HD camera for brightfield images and a Leica M205 FCA microscope equipped with a Leica DFC9000GT sCMOS camera for fluorescence images.

### Statistical analysis

Statistical analyses were carried out using GraphPad Prism 6 and 7. All data were first analysed for normal distribution using Shapiro–Wilk normality tests before applying tests for statistical significance. Likely outliers were identified by ROUT tests. For all tests the α level was set to 0.5. Circles in graphs represent individual experiments. The red line shows the median if not otherwise indicated. In case of parametric distribution graphs may show means ± SEM and circles indicating individual experiments. Embryo numbers are given within the figures, usually below the respective bars or scatters. Please see section about “zebrafish handling” and “immunofluorescence” for information about group sizes.

## Supplementary information

Supplementary Information

## Data Availability

All data generated or analysed during this study are included in this published article (and its supplementary information files).
