# Supplementary material for: Pharmacological cholesterol depletion disturbs ciliogenesis and ciliary function in developing zebrafish
Source: Commun Biol. 2019 Jan 29;2:31. doi: 10.1038/s42003-018-0272-7 (PMC6351647; doi:10.1038/s42003-018-0272-7)
Supplement: Supplementary file 1 — Supplementary Information [file 42003_2018_272_MOESM1_ESM.pdf]

SUPPLEMENT

Maerz LD *et al*.

SUPPLEMENTARY FIGURES WITH LEGENDS

Supplementary Figure 1: Cholesterol supplementation rescues statin-induced heart phenotype

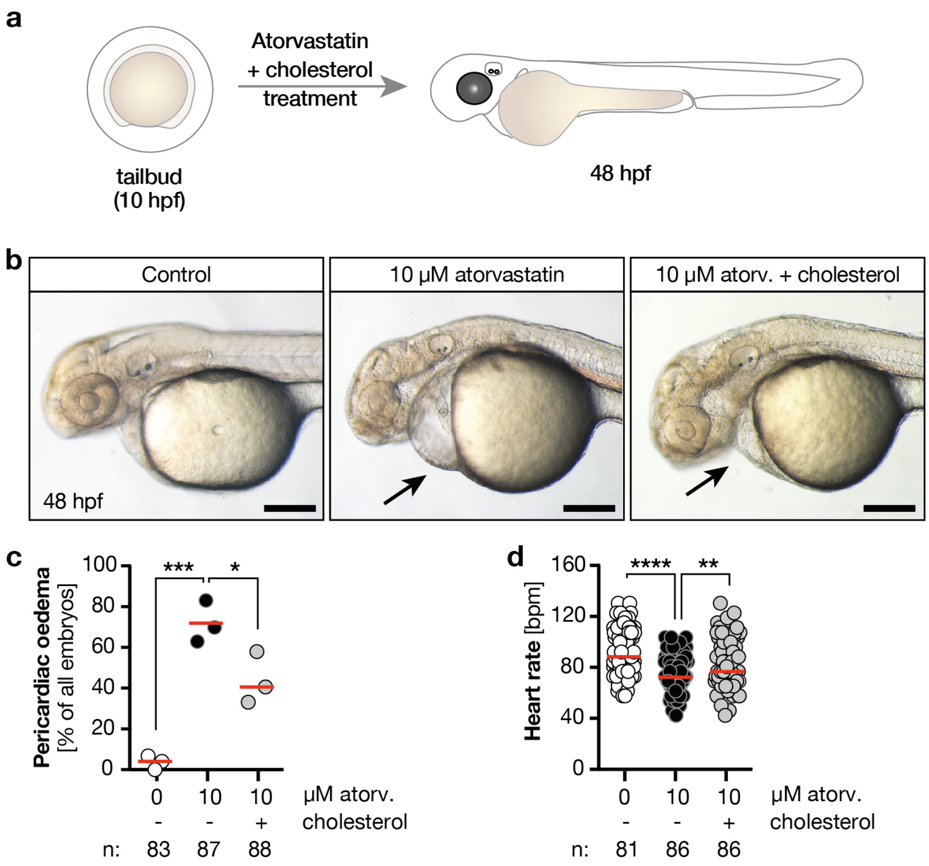

a, Zebrafish embryos were treated from tailbud stage until 48 hours post fertilization (hpf) with atorvastatin and water-soluble cholesterol.

b, Live images of embryos treated either with vehicle (1% DMSO in egg water, Control), 10 µM atorvastatin or 10 µM atorvastatin together with 10 µM cholesterol. Arrows indicate pericardiac cavity, which is less dilated upon cholesterol reconstitution. Scale bar: 200 µm.

c, Formation of pericardiac oedema is partly rescued after cholesterol supplementation. n=3 experiments. * p=0.0239, *** p=0.0003. One-way ANOVA with Sidak's post-test.

d, Heart rate significantly increases by co-administering cholesterol to atorvastatin-treated fish. n=3 experiments, each circle is one embryo.. ** p=0.0056, **** p<0.0001. Kruskal-Wallis test with Dunn's multiple comparison test.

Red line: median. Number of embryos given below graphs.

Supplementary Figure 2: Treatment with other statins phenocopies atorvastatin phenotypes

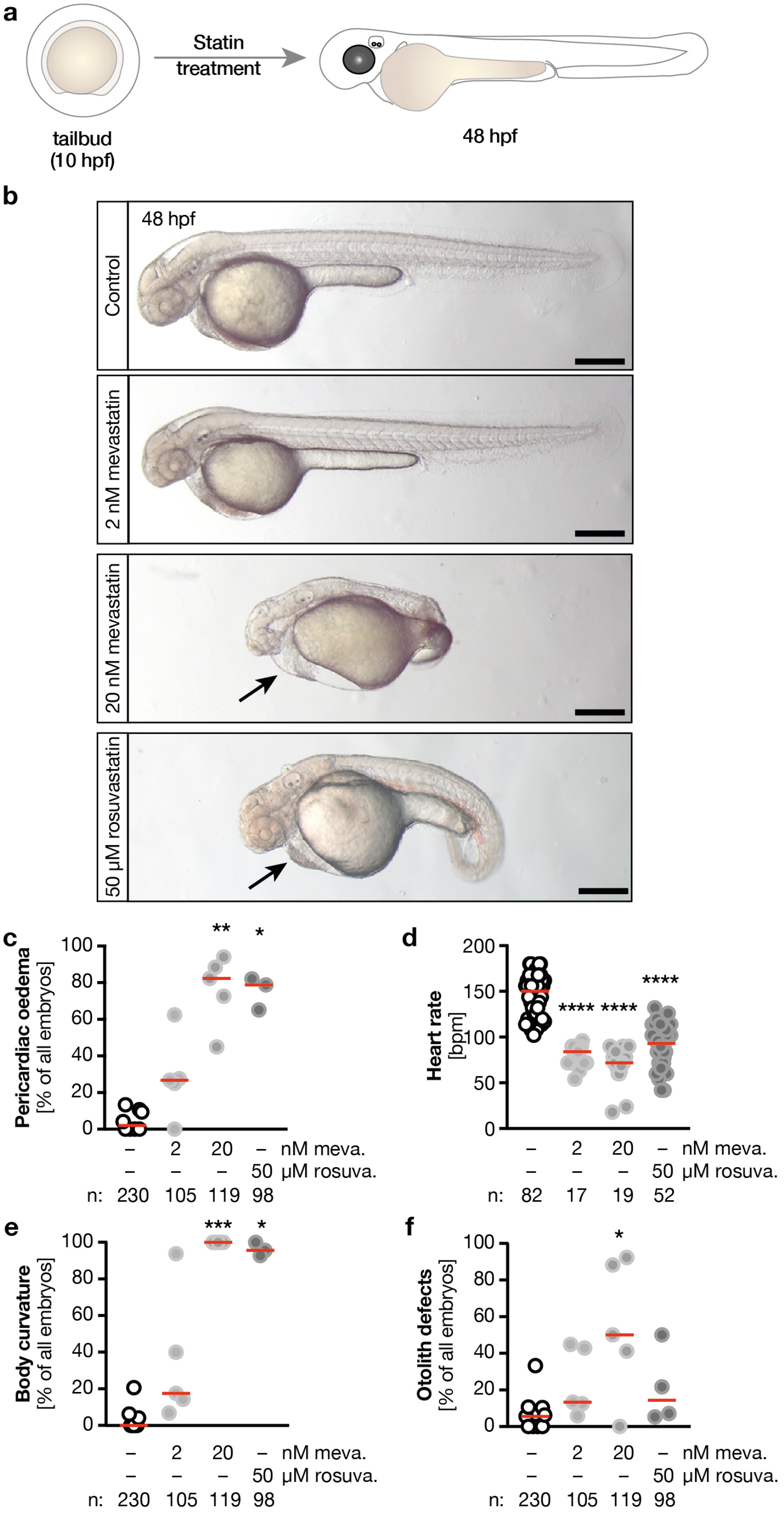

a, Zebrafish embryos were treated from tailbud stage until 48 hpf with mevastatin or rosuvastatin.

b, Live images of embryos treated either with vehicle (1% DMSO in egg water, Control), 2 or 20 nM mevastatin and 50 µM rosuvastatin. Arrow indicates pericardiac oedema. Scale bar: 200 µm.

c, The formation of pericardiac oedema upon statin treatment. * p=0.0187, ** p=0.0011.

d, Heart rate decreases with statin treatment. **** p<0.0001.

e, Meva- and rosuvastatin produce curved bodies. * p=0.0300, *** p=0.0003.

f, Meva- and rosuvastatin treatment also results in otolith defects.
* p=0.0309.

Number of embryos given below graphs. Red line indicates median.

c, **e** and **f**, number of experiments: 9 (DMSO), 5 (meva.) and 4 (rosu.)

c-f, Kruskal-Wallis test with Dunn's multiple comparison test. Statin vs. respective statin.

Supplementary Figure 3: Cholesterol replenishment partially rescues atorvastatin-induced ciliopathy phenotypes

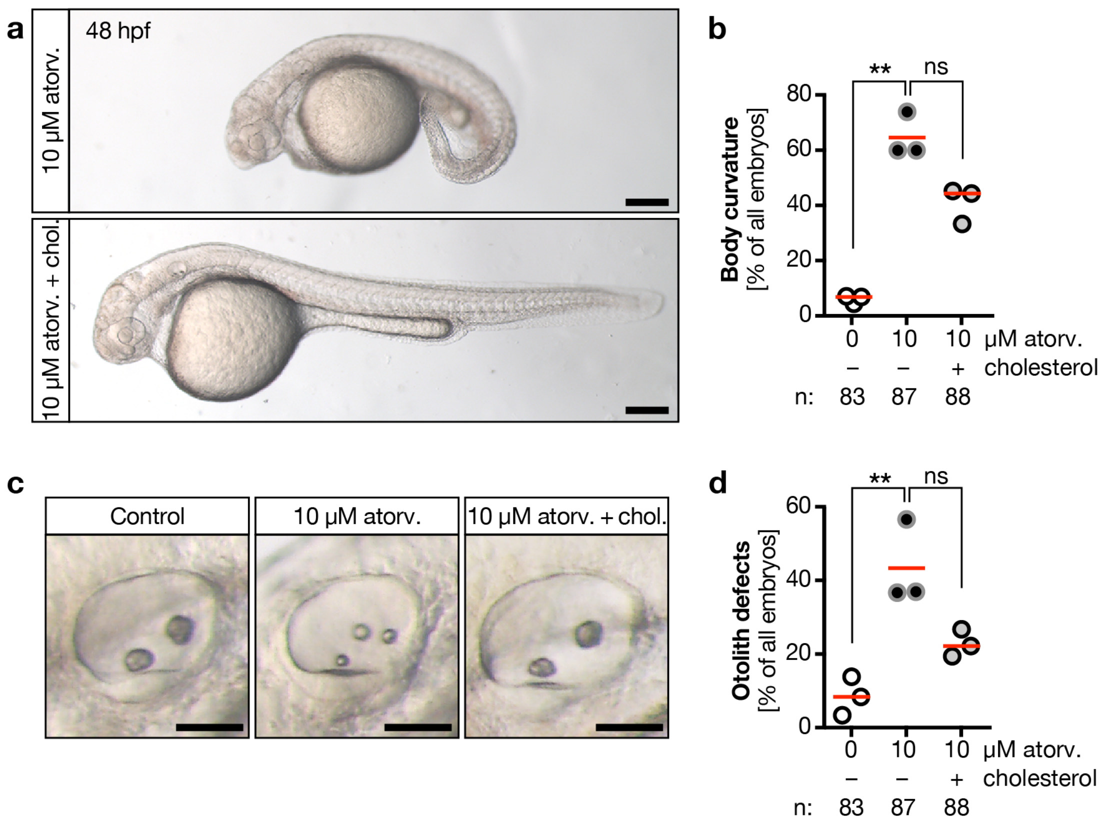

a, Atorvastatin treatment results in embryos with curved bodies, which can be rescued by cholesterol. Live images at 48 hpf. Scale bar: 200 µm.

b, Body curvature worsens by atorvastatin treatment and is improved upon cholesterol replenishment. Red line: median. n=3 experiments. *** p=0.0044, ns=0.0824, Kruskal-Wallis test with Dunn's multiple comparison test.

c, Live images of otic placodes at 48 hpf showing defects in otolith formation in atorvastatin treated embryos and regular otolith seeding in embryos co-treated with cholesterol. Scale bar: 50 µm.

d, Graph displaying the percentage of otolith defects upon atorvastatin and concomitant cholesterol treatment and. Red line: median. n=3 experiments. * p=0.0295, ns: p=0.3125, Kruskal-Wallis test with Dunn's multiple comparison test.

Supplementary Figure 4: Other statins induce left-right asymmetry defects, too

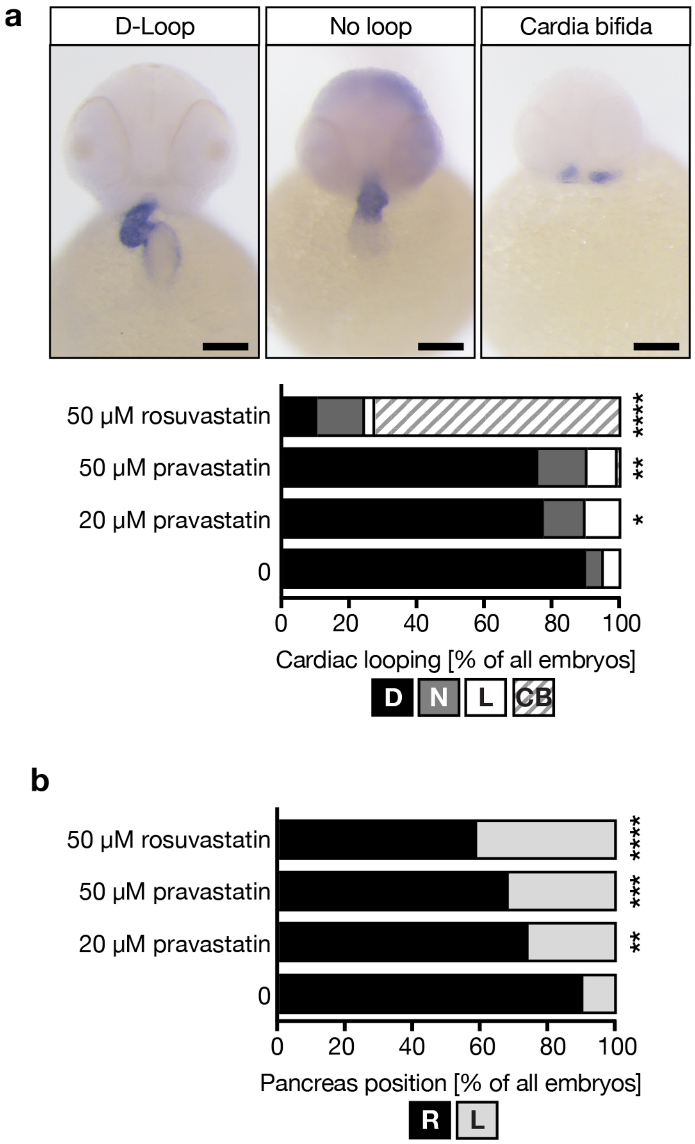

a, Pravastatin and rosuvastatin interfere with heart looping. In addition, cardia bifida could be observed. In situ hybridization for *cardiac myosin light chain 1* (*cmlc2*). Scale bar: 100 μm. *p=0.0165 (20 μM prava.), **p=0.0087 (50 μM prava), ****p<0.0001. Two-tailed Fisher's exact tests (all compared to DMSO-treated controls).

b, Pancreas placement is also randomized by treatment with other statins. **p=0.0023, ***p=0.0001, ****p<0.0001.

Stacked bar graphs summarize data of 4 experiments with 123 (DMSO), 97 (20 μM pravastatin), 90 (50 μM pravastatin) and 91 (50 μM rosuvastatin) embryos.

Supplementary Figure 5: Statin treatment during LR asymmetry determination is sufficient to produce ciliopathy defects

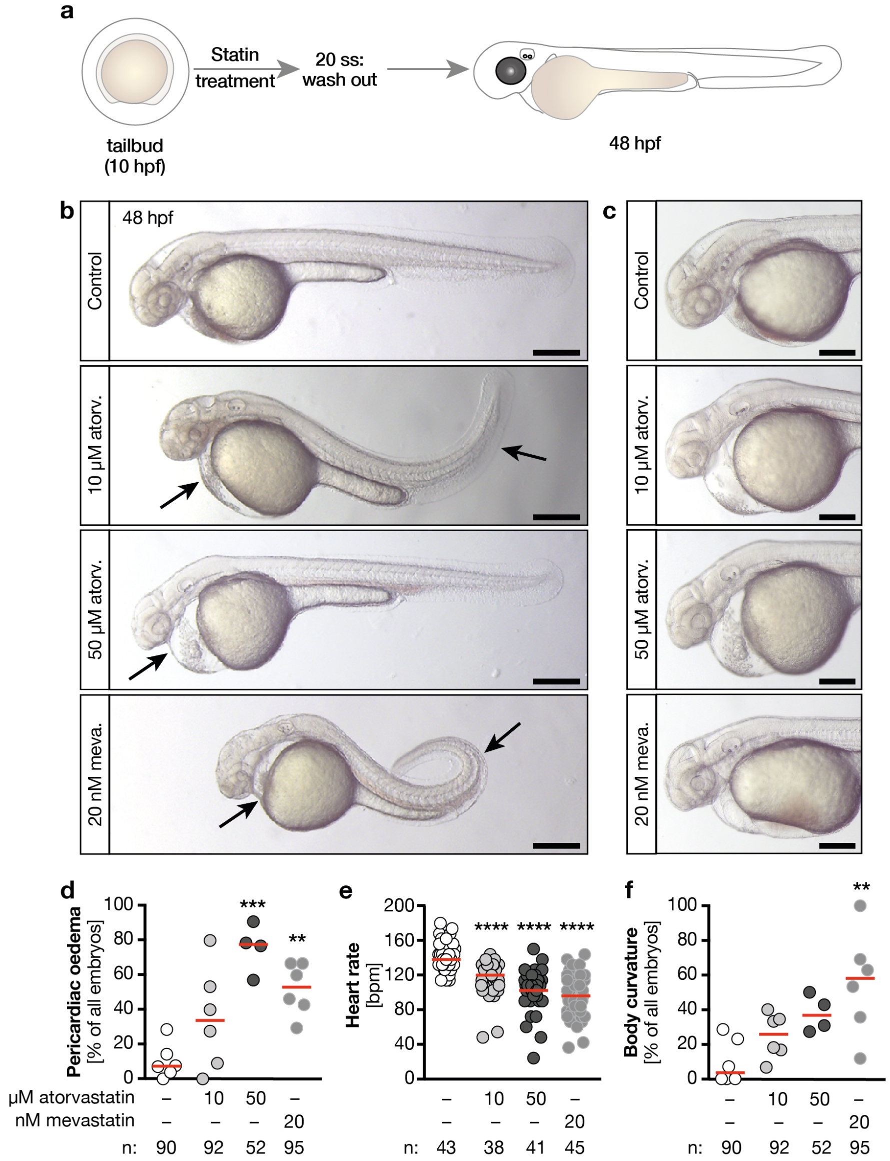

a, Zebrafish embryos were treated from tailbud stage until the 20 somites stage (ss) with atorvastatin or mevastatin. After washout of the drugs embryos were incubated until the 48 hpf stage.

b, Live images of 48 hpf embryos treated either with vehicle (1% DMSO in egg water, Control), 10 or 50 µM atorvastatin or 20 nM mevastatin. Arrows indicate pericardiac oedema and curved tails. Scale bar: 200 µm.

c, Live images of pericardiac oedema in higher magnification. Scale bar: 200 µm.

d, The formation of pericardiac oedema increases with atorvastatin and mevastatin treatment. n=6 (DMSO, 10 µM atorv., 20 nM mevastatin) and 4 (50 µM atorv.) experiments. ** means $p<0.01$, *** indicates $p<0.001$. One-way ANOVA with Bonferroni's multiple comparisons test.

e, Heart rate is also decreased when statins are only administered during left-right (LR) asymmetry cilia stages. **** $p<0.0001$, Kruskal-Wallis test with Dunn's multiple comparison test.

f, Mevastatin produces curved bodies. n=6 (DMSO, 10 µM atorv., 20 nM mevastatin) and 4 (50 µM atorv.) experiments. ** indicates $p<0.001$, Kruskal-Wallis test with Dunn's multiple comparison test.

d - **f**, circles: individual embryos (e) or experiments (d, f), red line: median.

Number of embryos given below graphs.

Supplementary Figure 6: Statin treatment results in shorter neural tube cilia

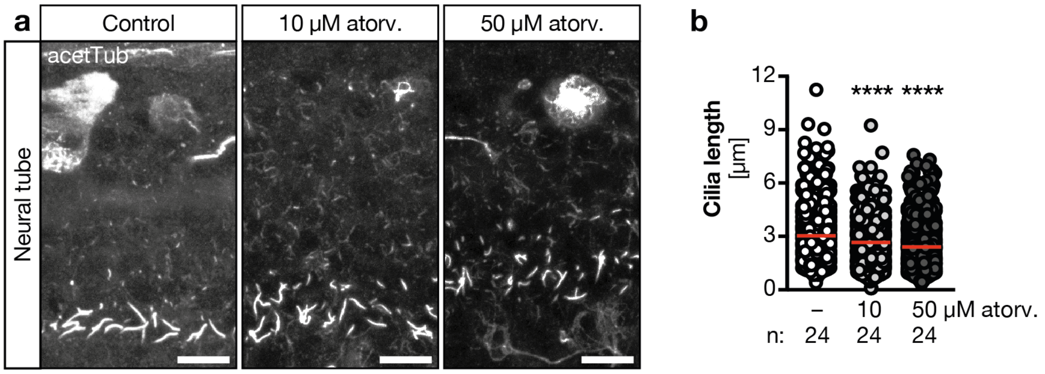

a, Confocal stacks of zebrafish neural tubes at 24 hpf. Cilia were labelled by antibody staining against acetylated tubulin (acetTub). Scale bar: 10 µm.

b, Atorvastatin decreases the length of cilia in the neural tube. Circles: individual cilia, red line: median. **** p<0.0001, Kruskal-Wallis test with Dunn's multiple comparison test.

Number of embryos given below graphs.

Supplementary Figure 7: Atorvastatin provokes kidney defects

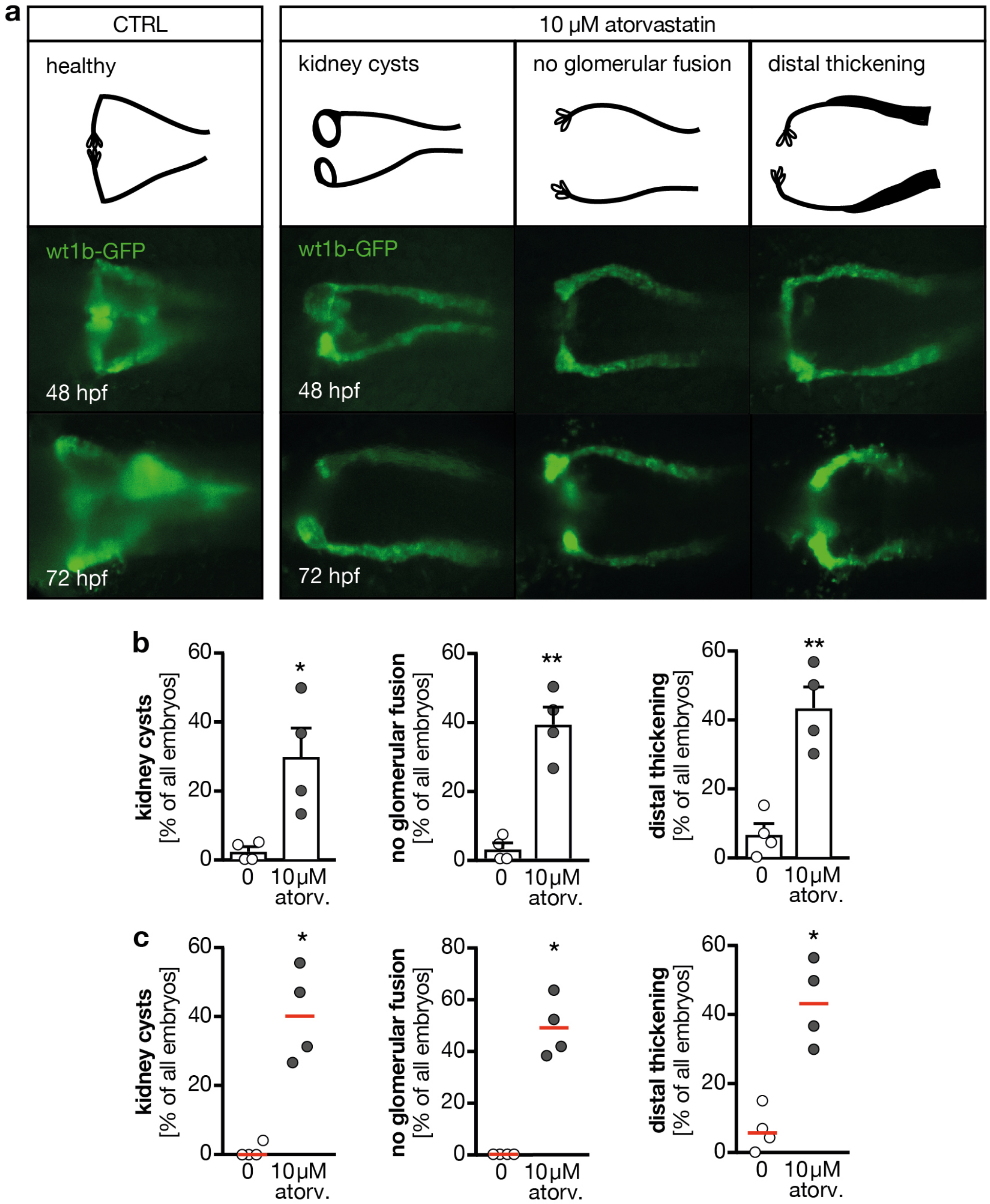

a, Treatment with atorvastatin from tailbud stage on results in kidney cysts, impaired glomerular fusion and distal thickening of the pronephric tubules. Fluorescence images of Wt1-GFP embryos at 48 and 72 hpf after DMSO control treatment or atorvastatin administration.

b, Bar graphs summarizing the incidence of kidney cysts, impaired glomerular fusion and distal thickening at 48 hpf. *p=0.0425, **p=0.0032 (no glomerular fusion) and 0.0041 (distal thickening).

c, Bar graphs summarizing the incidence of kidney cysts, impaired glomerular fusion and distal thickening at 72 hpf. *p=0.0286 (all three parameters).

All graphs: n=4, means ± SEM. two-tailed t-test with Welch's correction.

Supplementary Figure 8: Cilia length of *Tetrahymena thermophila* is altered by atorvastatin treatment

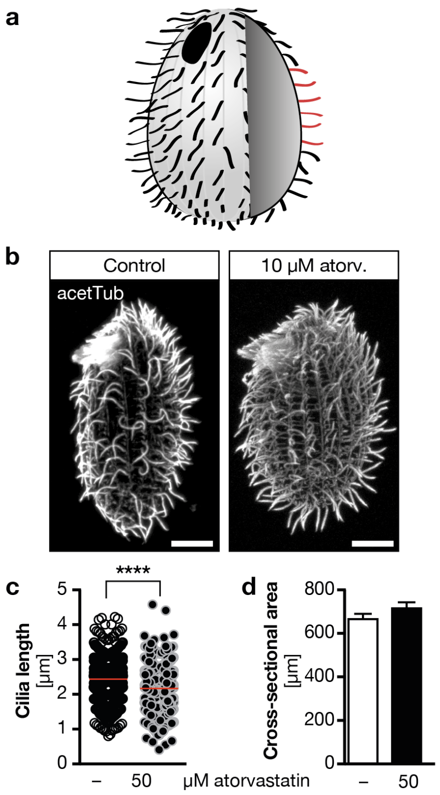

a, Cartoon showing a *Tetrahymena thermophila* cell. The oral apparatus is on the top left. Cilia were measured only in the mid-region of the cell (indicated in red).

b, Confocal stacks of *Tetrahymena*. Cilia were labelled by antibody staining against acetylated tubulin (acetTub). Images were selected based on cilium length differences. In all treatment groups great variation regarding cilia density could be observed. This did not appear to depend on cholesterol depletion, but can also be seen in untreated wild-type cells. Scale bar: 10 µm.

c, Atorvastatin decreases cilia length. n=6 experiments with 590 (DMSO) and 569 (50 µM atorv.) cilia of 59 (DMSO) and 58 (50 µM atorv.) cells in total. Circles: individual cilia, red line: median. $p<0.0001$, two-tailed Mann-Whitney test.

d, The cross-sectional area of *Tetrahymena* is not altered upon atorvastatin treatment. Means ± SEM. n=3 experiments with 24 embryos in total. $p=0.1170$, two-tailed t-test with Welch's correction.

Supplementary Figure 9: Atorvastatin administration does not alter cell size

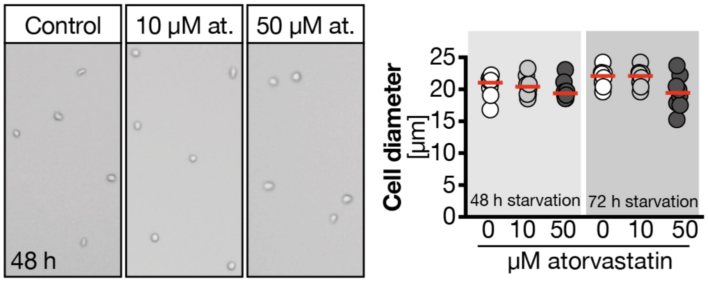

Fibroblasts treated with atorvastatin during 48 or 72 hours of starvation do not show significant changes in cell size. Live images of trypsinised fibroblasts. Bar graphs displaying the cell size of ciliated fibroblasts after two and three days of serum starvation. n=8. Red line indicates median. Kruskal-Wallis test with Dunn's multiple comparisons test. p values (atorv. vs. vehicle): >0.999 (48 h, 10 and 50 µM), >0.999 (72 h, 10 µM), 0.1249 (72h, 50 µM).

Supplementary Figure 10: Zebrafish treated with atorvastatin are impaired in Hedgehog signaling

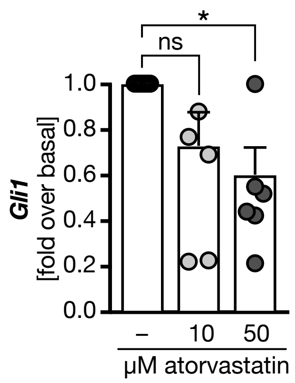

Hedgehog target gene expression is reduced in atorvastatin-treated zebrafish embryos. n=7 experiments. Means ± SEM with circles indicating individual experiments. p-values: 0.1154 (DMSO versus 10 µM atorv.), 0.0309 (DMSO versus 50 µM atorv.). Paired One-way ANOVA.

Supplementary Figure 11: Atorvastatin disrupts endothelium formation in air-liquid-interface (ALI) cultures

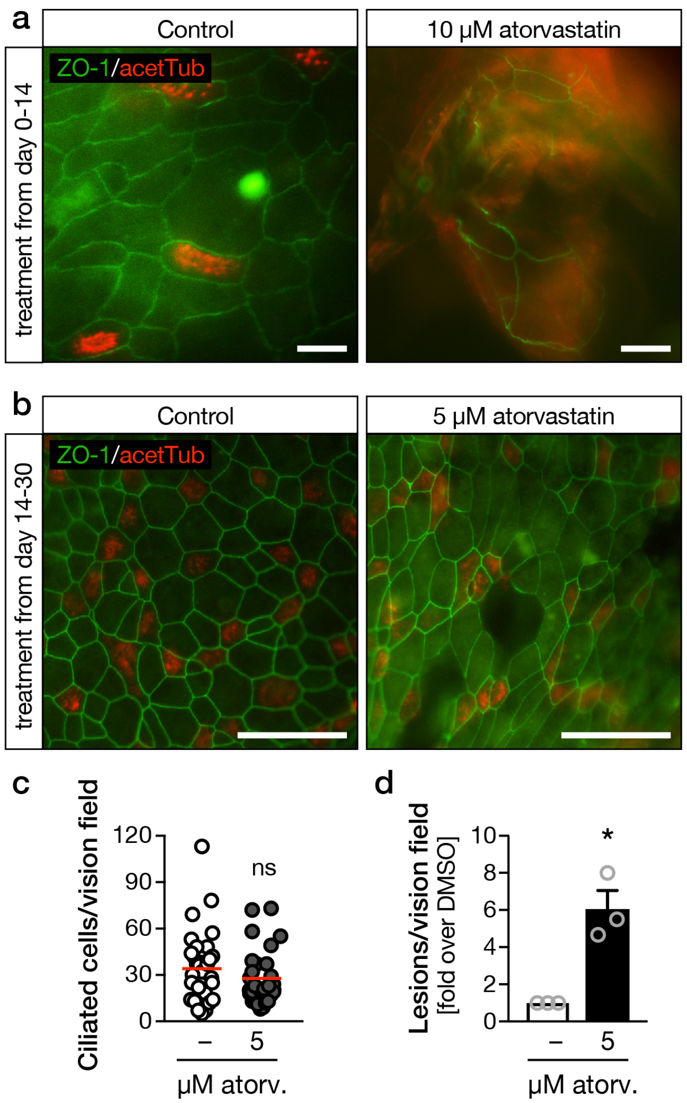

a, Atorvastatin treatment at the onset of human tracheal epithelial cell differentiation into ALI prevents formation of a continuous epithelium. n=5. Cilia were stained with acetylated tubulin (acetTub, red), apical cell borders with ZO-1 (green). Scale bar: 50 µm.

b, Atorvastatin treatment from day of human bronchial epithelial cell differentiation into ALI does not prevent ciliation, but results in a discontinuous epithelium. n=3. Cilia were stained with acetylated tubulin (acetTub, red), apical cell borders with ZO-1 (green). Scale bar: 50 µm.

c, Atorvastatin treatment did not alter the number of ciliated cells per vision field. n=3 with 10 vision fields per condition. p=0.1292, two-tailed Mann-Whitney test.

d, Atorvastatin induces more lesions compared to DMSO-treated control cultures. n=3, means ± SEM. p=0.0371, two-tailed t-test with Welch's correction.

Cropped western blots of Figure 4:

P-AKT

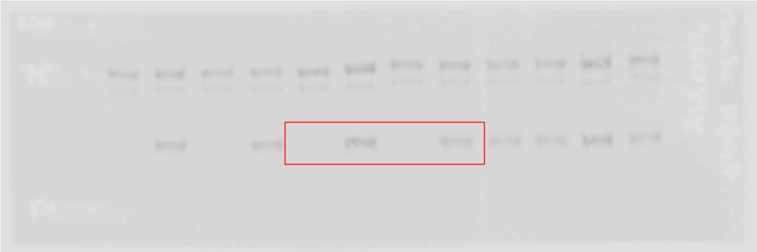

AKT

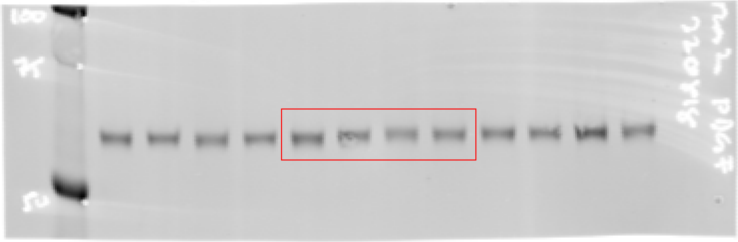

Gapdh

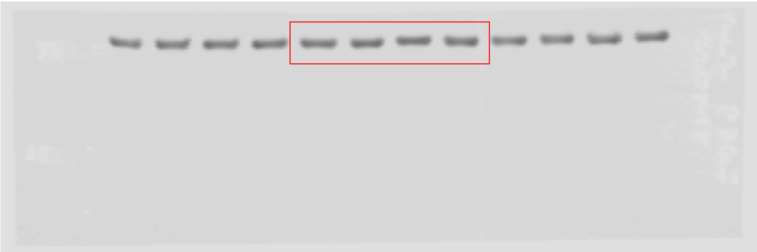

Cropped western blots of Figure 5

P-AKT

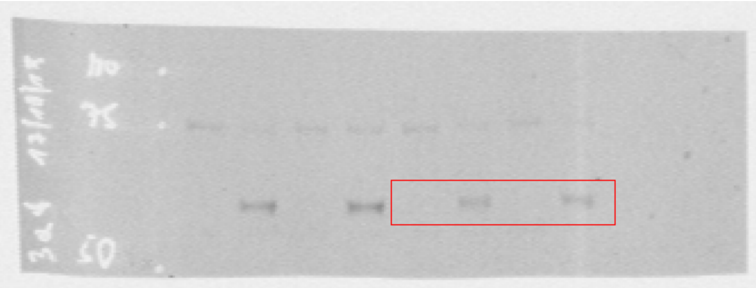

AKT

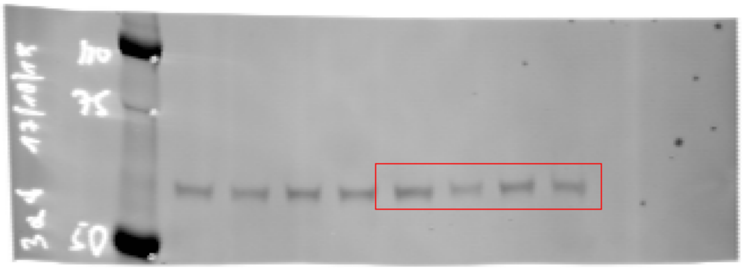

Gapdh

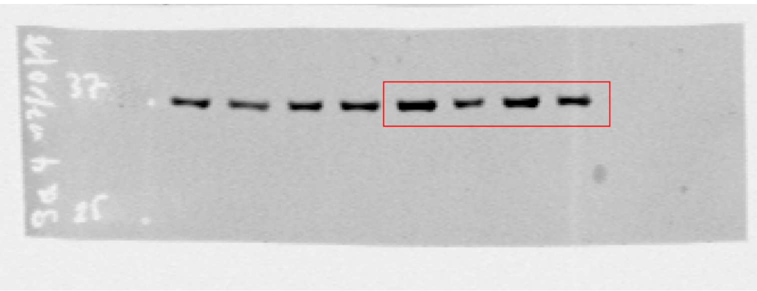

SUPPLEMENTAL METHODS

Tetrahymena treatment

Tetrahymena thermophila stocks (wild type strain CU428, kindly provided by Dorota Wloga, Nencki Institute of Experimental Biology, Poland) were maintained in 5 ml SPP medium (1 % peptone proteose, 0.2 % D+-glucose, 0.1 % yeast extract and 0.03 % EDTA-Fe3+ salt (all Sigma)) in 15 ml falcons at room temperature without shaking. The day before the experiment, experimental cultures were inoculated and grown to a density of $2x10^5$ cells/ml. 50 µM atorvastatin or DMSO was added to the culture medium and the cells incubated at 30 °C and 80 rpm in an orbital shaker for 24 hours.

Immunofluorescence

To stain *Tetrahymena* cells, 10 µl of cultures were spotted on a coverslip and 10 µl of Fix/Perm (8 % PFA and PHEM (60 mM PIPES, 25 mM HEPES, 10 mM EGTA, 2 mM $MgCl_2$) 1:1, supplemented with 1 % NP-40) was added. After drying the cells were blocked with 3 % bovine serum albumin in PBS for 10 min and incubated with primary antibody diluted in blocking buffer overnight at 4 °C. On the next day cells were carefully washed three times during 10 min with PBS and incubated with secondary antibody for 90 min at room temperature in the dark. Cells were mounted with Vectastain containing Dapi and the edges sealed with nail polish.

Fibroblast cell size measurement

Analysis of cell number, viability and size was done using the Countess II automatic cell counter (Life Technologies, Germany). According to the manufacturer`s protocol 10 µl of cells were diluted 1:1 with trypan blue to visualize viability and cell death. Images were automatically acquired by the counter.
